# Filled Elastomers: Mechanistic and Physics-Driven Modeling and Applications as Smart Materials

**DOI:** 10.3390/polym16101387

**Published:** 2024-05-13

**Authors:** Weikang Xian, You-Shu Zhan, Amitesh Maiti, Andrew P. Saab, Ying Li

**Affiliations:** 1 Department of Mechanical Engineering, University of Wisconsin-Madison, Madison, WI 53706, USA; wxian3@wisc.edu (W.X.); yzhan48@wisc.edu (Y.-S.Z.); 2 Lawrence Livermore National Laboratory, Livermore, CA 94550, USA; maiti2@llnl.gov (A.M.); saab2@llnl.gov (A.P.S.)

**Keywords:** elastomer, nanoparticle, constitutive model, reinforcement, the Mullins effect

## Abstract

Elastomers are made of chain-like molecules to form networks that can sustain large deformation. Rubbers are thermosetting elastomers that are obtained from irreversible curing reactions. Curing reactions create permanent bonds between the molecular chains. On the other hand, thermoplastic elastomers do not need curing reactions. Incorporation of appropriated filler particles, as has been practiced for decades, can significantly enhance mechanical properties of elastomers. However, there are fundamental questions about polymer matrix composites (PMCs) that still elude complete understanding. This is because the macroscopic properties of PMCs depend not only on the overall volume fraction (ϕ) of the filler particles, but also on their spatial distribution (i.e., primary, secondary, and tertiary structure). This work aims at reviewing how the mechanical properties of PMCs are related to the microstructure of filler particles and to the interaction between filler particles and polymer matrices. Overall, soft rubbery matrices dictate the elasticity/hyperelasticity of the PMCs while the reinforcement involves polymer–particle interactions that can significantly influence the mechanical properties of the polymer matrix interface. For ϕ values higher than a threshold, percolation of the filler particles can lead to significant reinforcement. While viscoelastic behavior may be attributed to the soft rubbery component, inelastic behaviors like the Mullins and Payne effects are highly correlated to the microstructures of the polymer matrix and the filler particles, as well as that of the polymer–particle interface. Additionally, the incorporation of specific filler particles within intelligently designed polymer systems has been shown to yield a variety of functional and responsive materials, commonly termed smart materials. We review three types of smart PMCs, i.e., magnetoelastic (M-), shape-memory (SM-), and self-healing (SH-) PMCs, and discuss the constitutive models for these smart materials.

## 1. Introduction

Rubbery materials are omnipresent in Mother Nature, with feature sizes ranging from the nanoscale to the macroscale. However, the understanding and further application of the materials remained limited until the invention of vulcanization treatment independently by Hancock [1] and Goodyear [2]. The most iconic application of vulcanization is the production of automobile tires. Despite the long history of applications of natural rubber, a fundamental understanding of the material began to take shape only in the early 20th century [3] with the first development of polymer chemistry and physics theories.

Curing (crosslinking) primarily involves crosslinking within the elastomeric matrix that leads to a mechanically stable network, while the incorporation of filler particles and other additives into the polymeric matrix yields composites with enhanced mechanical properties. Incorporation of filler particles into the polymeric matrix usually involves physical processes. Crosslinking can be categorized into chemical and physical types. An example of chemical crosslinking is sulfur vulcanization applied to natural rubber (NR). Connections between the matrix chains are formed by sulfur, usually under heated conditions with the help of an activator and accelerator that activates and accelerates the crosslinking reaction, respectively. Wide applications of elastomers in various industries, such as automotive, aerospace, and structural engineering, would be impossible without these two approaches of reinforcement [4]. Although different techniques of crosslinking and filler reinforcement have been empirically developed in industries, gaining a better understanding of the microscopic mechanism of the reinforcements is an active field of research. Such knowledge is highly valuable not only from a scientific standpoint but is also of great importance in guiding the design of future materials.

Elastomers are synthesized from polymer precursors that are sourced from natural substances or synthetic products. Polymer molecules, regardless of the topology such as linear, branched, comb-like, ring-like, or star-like, are joined chemically by the crosslinking process to form a percolated network. Although the crosslinking mechanism can be diverse, e.g., covalent reactions or physical associations, the crosslinking process leads to structural integrity of the network that dictates the enhanced load-bearing capabilities [1,2]. In general, the mechanical strength of the elastomers is correlated to the number density of the crosslinking chain segments, termed crosslink density [5]. However, a technological dilemma is that the extensibility of the elastomer decreases as the crosslink density increases. As an example, epoxy is a polymeric material of extremely high crosslink density. The resulting high stiffness and low extensibility make epoxies very brittle [6].

The earliest effort to explain the reinforcement effects introduced by micro- or nanoscale filler particles dates back to the hydrodynamics effect proposed by Einstein [7]. In this model, it is assumed that the particles are isolated and distanced from each other such that their influence on the adjacent polymer matrix is only local. While such a theory is reasonably applicable to composite systems with low-to-moderate loading of micron-sized filler particles, it significantly underestimates the mechanical strength of the composite systems when the loading of particles is high and when the intrinsic size of the particles is smaller than micron scale [8]. Except at very low loading levels, the modulus of the composites usually increases nonlinearly as a function of particle loading. Such nonlinear reinforcement is complex to understand, even at a qualitative level. First, it is attributed to the possibility that the particles form additional network structures at the micro- or mesoscale because of the high surface-to-volume ratio of the filler particles. Second, high particle loadings can lead to macroscopically large, percolated filler cluster arrangements that can have a significant contribution to the load-bearing capabilities of the composites [9]. Additional complexity results from the fact that the mechanical behaviors of the particle networks are different from those of the polymer networks because of the very different chemical constituents the two networks consist of. While the elasticity of the matrix networks is primarily entropic, the strength of the particle networks derives from the energetic interaction between the particles and across the particle–polymer interface. The particles are generally treated as rigid objects because the moduli of the filler particles, like carbon black and silica, are much higher than the matrix polymers. The structural and rheological properties of the polymers in and near interfaces can be notably different from those within the bulk of the matrix polymer [10], which can greatly affect the macroscopic properties of the composites [11]. For example, the transition from the melt state to the glass state is significant within the particle–polymer interface. The two states are associated with very different mechanical properties [12]. Additionally, given that polymer–particle interactions can be tuned by surface modification of the particles, with either hydrophilic or hydrophobic groups, different strategies have been developed to control the polymer–filler microstructure and the resulting mechanical properties of the composites [13].

It is worth noting that the reinforcement of the polymer matrix composite is a multiscale physical phenomenon. While the smallest characteristic scales associated with the corresponding Kuhn segments of the matrix polymers are at the molecular level, the scales can be orders of magnitude larger for the composite because of the network-like structures of the matrix polymers and the particles. However, the multiscale nature is not fully understood because there are still notable gaps between different temporal and spatial scales [14,15]. While the communities of mechanics and engineering may prioritize establishments of constitutive relationships to understand the macroscopic mechanical properties of the composites, polymer physicists and chemists may pay more attention to the microscopic structural and dynamics properties. Bridging knowledge across the spectrum of scales remains challenging but efforts have been made using different scientific and engineering approaches.

We present here an overview of recent progress in the understanding of reinforcement in PMCs. We aim to review not only works that delve into microscopic characteristics of composite elastomers, but also studies that focus on constitutive modeling approaches aimed at building a connection between microstructure and macroscopic properties. The rest of this paper is organized as follows. Commonly used materials for the matrix polymer and filler particles are briefly discussed in Section 2. Section 3 discusses notable constitutive models for the PMCs with details related to their treatments of reinforcement, hyperelasticity, viscoelasticity, the Mullins effect, strain-induced crystallization, and the Payne effect. Smart (or intelligent) materials are substances or composites that can respond to changes in their environment to achieve designed functionalities. The external stimuli include temperature, light, pH, electric or magnetic fields, mechanical stress, and chemical compounds [16]. As smart materials have drawn great attention because of their capability and versatility, we review applications of filled systems as smart materials in Section 4, including magnetoelastic, shape-memory, and self-healing systems. This is followed by a brief discussion and conclusions in Section 5.

## 2. Materials

There is a large collection of polymer and particle materials that have been studied for PMCs. However, the number of polymers that are usually used as matrices for the filled elastomers is limited for two reasons. Firstly, the costs of syntheses of these materials are critical, especially for industrial applications. The materials must be produced with a relatively easy synthesis route or be naturally available. The second reason is that it must be feasible to mix the materials. Industrial applications often rely on standard processing techniques of which small changes cost huge investments [17].

### 2.1. Polymer Matrix

Cis-1,4-polyisoprene (NR) had been the primary type of rubber material until World War I led to a significantly increased demand for rubber and resulted in the development of synthetic rubbers [18]. Polychloroprene (Neoprene) was the first commercially available synthetic rubber [19]. Poly(styrene-butadiene) or styrene-butadiene rubber (SBR) is another synthetic rubber that is used as a tire material due to its good resistance to wear. Not surprisingly, polybutadiene (PB) [20] and polystyrene (PS) [21] are also good rubber materials for many applications. Nitrile rubber (NBR) is a synthetic copolymer of acrylonitrile and butadiene with excellent resistance to alkalines and oil [22]. Silicone rubber (primarily polydimethylsiloxane) has also been widely used because of its inert properties such as high resistance to aging under extreme temperatures [23,24]. In recent years, hydrogels have also been used in many biomedical and coating applications. Alginate hydrogel is derived from natural substances like seaweed and is most commonly used in biomedical applications such as cell culture, drug delivery, and wound treatment [25]. Synthetic polymers for hydrogels include polyacrylamide [26], polyethylene glycol [27], and polyvinyl alcohol [28].

### 2.2. Filler Particle

**Figure 1:**
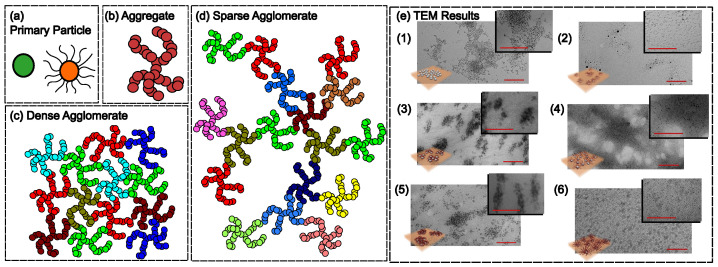
Schematic for the filler particles. Different colors represent individual nanoparticles or aggregates. (**a**) Spherical primary particles with and without surface modification. The size of primary particles ranges from 5 to 50 nm (**b**) An aggregate formed by the primary particles fused together. The size of an aggregate is about 100 nm. (**c**,**d**) Respective illustrations for dense and sparse agglomerates formed by aggregates by weak forces. The size of agglomerates is usually at least ∼100 nm, depending on interaction between the forming aggregates. (**e**) TEM results of NPs in polymer matrix. Adapted from [38] with permission from Elsevier Copyright (2019). The polychloroprene-grafted NPs are dispersed in the polychloroprene matrix differently according to their surface modifications. (1) Bare silica nanoparticles; (2) Medium-length chains on the particles with high graft density; (3) Short chains on the particles with low graft density; (4) Long chains on the particles with low graft density; (5) Short chains on the particles with high graft density; (6) Ultra-long chains on the particles with high graft density. Red lines are 200 nm scale bars.

The most widely used filler particles are carbon black (CB) because of the huge annual consumption in the tire industry [17]. Another commonly employed filler material is silicon dioxide (silica) particles because they outperform CB in improving several performance aspects of tire products. Particles with a size above $10^{3}$ nm are considered non-reinforcing because they are much larger than the size of polymer molecules and their surface-to-volume ratio is low. When the size of the particles approaches ∼$10^{3}$ nm or below, the reinforcement effect becomes pronounced because of the significant increase in the surface exposed to the matrix polymers, especially for the particles smaller than about $10^{2}$ nm [29,30]. Specific surface area, which is related to the size and shape of the filler particles, is also an important property, as it is one of the key factors that enhances the interaction between the matrix polymers and the filler particles [31]. Adsorption of gas molecules on a solid surface, as can be described by Brunauer–Emmett–Teller (BET) theory, is usually used to characterize the specific surface area of nanomaterials [32,33]. Nanometer-scale (diameter ∼$10^{2}$ nm or less) silica particles are usually manufactured by pyrogenic processes, because of which they are also termed fumed silica [34]. The size of particles changes as they experience temperature gradients during the pyrogenic processes. Primary particles (essentially a spherical shape) have sizes ranging from 5 to 50 nm, as in Figure 1a. Special treatments are needed to obtain stand-alone nanoparticles (NPs) of such sizes. Without these special treatments, the primary particles tend to fuse and form aggregates of size about $10^{2}$ nm or larger, as in Figure 1b. As the temperature during the pyrogenic processes decreases, the growth of the aggregates stops because of the lack of driving thermal energy. Separation of primary particles in the aggregates is difficult as it requires breakage of chemical bonds such that the aggregates are usually considered rigid bodies. However, due to the high surface-to-volume ratio, different aggregates can interact with each other via weak forces like van der Waals. Therefore, silica particles are usually in the form of agglomerates, unless high input energy is used to break them into isolated aggregates. The shape of the agglomerates depends on specific processing procedures, but it is usually fractal in nature. They can be both densely and sparsely packed, as shown in Figure 1c,d. Both NPs and aggregates of primary particles will be termed particles unless stated otherwise. The fractal characteristic is universal to most of the particles of nanometer size.

Interaction between particles can be changed by surface modification of the particles, as pictured in Figure 1a. Different molecules or functional groups can be linked chemically to the surface such that the particles can become more hydrophilic or hydrophobic depending on the specific chemistry. For example, a silane coupling agent, like 3-methacryloxypropyl trimethoxysilane, can be used to change a hydrophilic surface to hydrophobic via chemical bonding [35]. Such modifications are commonly used to tailor the interaction strength between the matrix polymer and the particles [13]. Polymer chains, rather than oligomers, can also be grafted to the particle surfaces through radical [36] or photocatalytic polymerizations [37] to improve the miscibility between the polymer matrices and the particles. In Figure 1e, different states of dispersion of silica NPs grafted with polychloroprene within a polychloroprene matrix are revealed by transmission electron microscopy (TEM) images [38]. As can be seen, the microstructures of the silica NPs can vary depending on the molecular weight of the modified molecule and density of the grafts, as well as on the concentration of the NPs, with examples such as: (1) bare NPs in low concentration; (2) mediate-chain grafted NPs with low grafted density in low concentration; (3) short-chain grafted NPs with low grafted density in mediate concentration; (4) long-chain grafted NPs with low grafted density in high concentration; (5) short-chain grafted NPs with high grafted density in mediate concentration; and (6) ultra-long-chain grafted NPs with high grafted density in mediate concentration.

**Figure 2:**
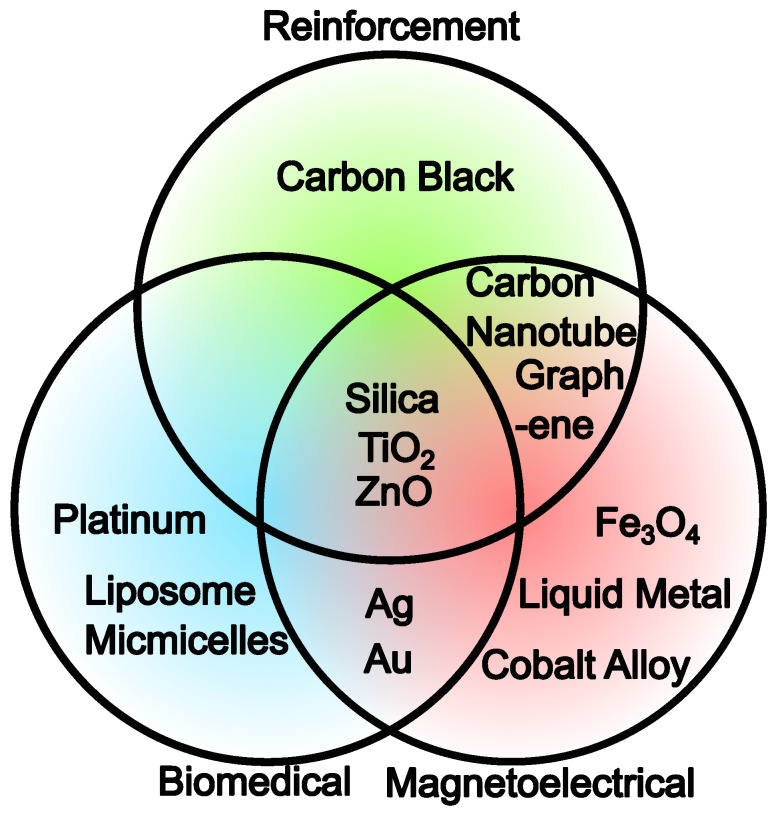
A summary of the commonly used filler particles.

TiO_2_ [39] and ZnO [40] are other common types of filler particles used in reinforcement. Filler particles are also incorporated into the polymer matrix for other functions. For example, gold and silver NPs are often employed in biomedical applications because of their antimicrobial attributes [41]. Fe_3_O_4_ particles are used as fillers in magnetic soft materials due to their ferromagnetic properties [42]. Non-particle-like materials have also found application as fillers. For instance, composites incorporating carbon nanotubes [43] and graphene [44], and graphite [45] have been widely recognized for their superior structural, mechanical, and even electrical properties while being lightweight. Liquid metals (LMs) constitute another intriguing filler system because the state of LMs can be tuned directly by external fields, which is utilized in the application of LM composites in diverse applications [46]. A graphical summary of commonly used filler particle systems is presented in Figure 2.

## 3. Constitutive Modeling

**Figure 3:**
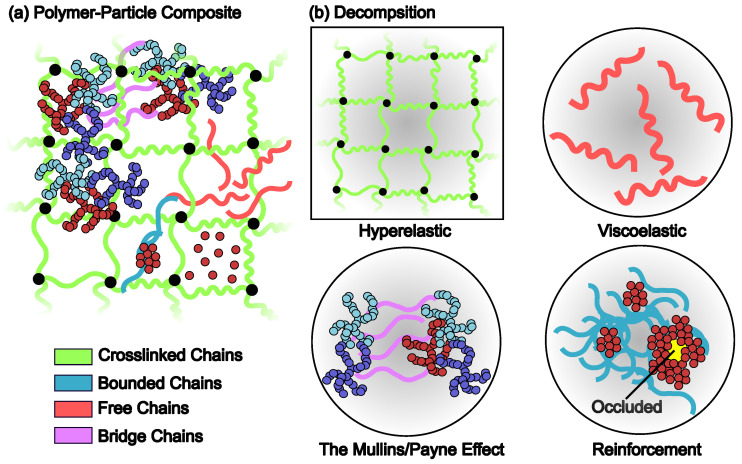
Decomposition in the constitutive model. (**a**) Schematic of polymer matrix composite (PMC). (**b**) Decomposition into the different contributions. Hyperelasticity originates from the crosslinked network. Viscoelastic behavior is attributed to the free chains, which can relax and diffuse. Other inelastic effects like the Mullins effect and the Payne effect are highly related to the bridging chains that connect different particles and aggregates. Bounded chains that form a glassy interface can overlap when the particle loading ϕ is high. In the extreme case, occluded volume with enclosed chains can be formed.

**Figure 4:**
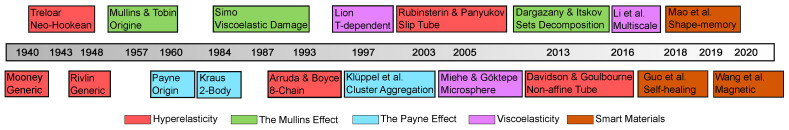
Timeline of selected constitutive models regarding the hyperelasticity, the Mullins effect, the Payne effect, and smart materials.For the hyperelasticity, literatures include: Mooney [48], Treloar [49], Rivlin [50], Arruda and Boyce [51], Rubinstein and Panyukov [52], and Davison and Goulbourne [53]. For the Mullins effect, literatures include: Mullins and Tobin [54], Simo [55], and Dargazany and Itskov [56]. For the Payne effect, literatures include: Payne [57], Kraus [58], and Klüppel et al. [59]. For the viscoelasticity, literatures include: Lion [60], Miehe and Göktepe [61], and Li et al. [62]. For the smart materials, literatures include: Guo et al. [63], Mao et al. [64], and Wang et al. [65].

Constitutive models are designed to describe macroscopic mechanical behavior, attempting to connect microscopic and molecular-level properties of the material to its bulk properties of the continuum. Constitutive models typically involve defining an expression for free energy density, *W*. For many material systems, it has been well recognized that multiple individual physical mechanisms contribute independently to *W* [47], as depicted schematically in Figure 3a for PMCs. Generally, filler particles can be in different dispersion states, as mentioned in the previous section. On the other hand, the state of the polymer chains can be categorized into several types, including (1) crosslinked chains that form the matrix of the elastomer; (2) bounded chains that are adsorbed at the surface of particles or their clusters/aggregates; (3) free chains that can diffuse within the PMCs; and (4) bridge chains connecting different filler particles. The decomposition of *W* into contributions from different types of chains accounts for the respective mechanical properties stated in Figure 3b. In the following sections, the relative importance of these contributions will be detailed. Figure 4 displays notable constitutive models that have been widely used to quantify the hyperelastic and inelastic behaviors of unfilled or filled elastomers. The list is arranged chronologically in order of the publication date.

### 3.1. Hyperelasticity

**Figure 5:**
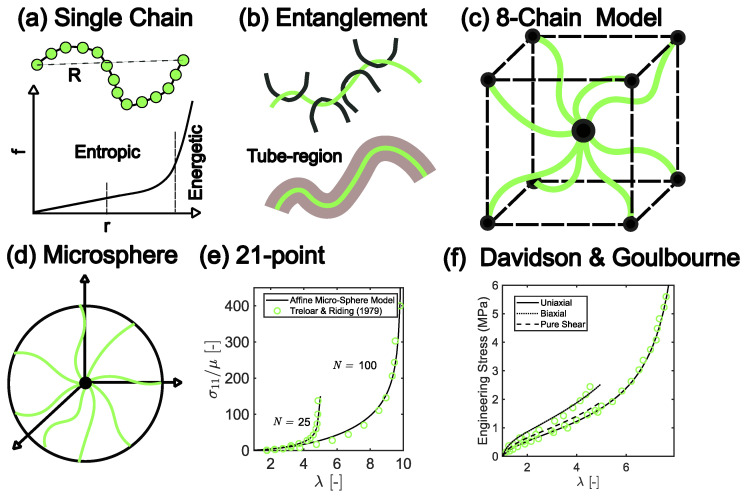
Hyperelastic mechanics of elastomer. (**a**) Single-chain behavior includes three phases: linear entropic, nonlinear entropic, and energetic stretch regimes. (**b**) Entanglement, topological constraint, can be equalized as a tube-like region confining the center chain [68,69]. (**c**) Schematic for the 8-chain model. (**d**) Schematic for the microsphere model. (**e**) Stress–strain curves predicted by the 21-point scheme of the microsphere model [70] compared with experimental measurement from [71]. (**f**) Stress–strain curves predicted by the nonaffine tube model by Davidson and Goulbourne. Adapted from [53] with permission from Elsevier Copyright (2013). In (**e**,**f**), green circles are experimental data and black solid lines are model predictions.

One of the important physical characteristics of rubber-like solids is the ability to sustain finite deformation under a small amplitude of load. The second is that rubber materials can recover a deformed state with no or little hysteresis. To analyze the mechanical properties of rubber, constitutive models that capture the hyperelasticity characteristics are valuable. The microstructure of hyperelastic solids comprises a crosslinked network with the constituent chains randomly oriented. Description of the elastic free energy of such a molecular network using the principles of thermodynamics is at the heart of building a constitutive model. Therefore, tools of statistical mechanics are a natural choice in such model development. One classic statistical model for a single chain is the Gaussian chain description, in which the chain is modeled as a random walk with *N* segments of bond length *b*. Conformation of the chain is quantified by the instantaneous end-to-end vector *R*, with $\langle R\rangle =0$ and $\langle R^{2}\rangle =Nb^{2}$ the average size of the chain. The relation between amplitude of force *f* required to stretch the chain to separation r=|R| is linear if $r<<\sqrt{Nb^{2}}$. In this case, the elasticity is entropic. In the regime where *r* increases and approaches the contour length, Nb, of the polymer chain, the number of permissible conformations for the chain decreases dramatically. This causes an increase in the chain stiffness, which can be described by Langevin chain statistics [66]. It is worth noting that *b* remains unchanged in both regimes. Stretching the chain beyond its contour length causes an energetic stretch of *b* until the bond breaks. Thus, as the chain length, *r*, increases there is an entropic-to-energetic transition in the single chain *f* behavior [67], as shown in Figure 5a. Mechanistic models have been developed to cover this physical picture. Another important characteristic of rubber materials is that entangled polymer chains cannot cross each other, as illustrated in Figure 5b. Thus, for $N>N_{ent}$, the topological non-crossability constraint suppresses the relaxation of the chains. This, in addition to the crosslinking effect, changes the mechanical properties. Here, $N_{ent}$ is the threshold chain length for entanglement, and it is concentration-dependent [68,69].

Early attempts to model the hyperelastic behavior of elastomers were built upon phenomenological approaches that ignore the statistical description alluded to above. Such phenomenological models primarily rely on mathematical reasoning, aiming at finding generic ways to characterize the experimental observed stress–strain response with calibrated empirical parameters. At the most fundamental level, all phenomenological models make use of an important mathematical result called representation theory, according to which the strain energy density function, *W*, of an isotropic system can be expressed as a function of the invariants, $I_{i}$ (*i* ranges from 1 to 3), of the left Cauchy–Green deformation tensor $B=FF^{⊤}$. F is the deformation gradient tensor that maps the configuration in reference state X to current state x. One of the earliest models to describe the finite stretchability of rubbers is the Mooney model [48]. With the assumption of incompressibility and isotropicity in the unconstrained state of the elastomer, *W* is expressed in terms of only two invariants, i.e., $I_{1}$ and $I_{2}$, as in Equation (1) where $C_{1}$ and $C_{2}$ are materials constants to be calibrated empirically from experiments.

(1) $$W=C_{1}\left(I_{1}-3\right)+C_{2}\left(I_{2}-3\right)$$

Another classic model with an even simpler functional form is the neo-Hookean model [49]. In this model, the strain energy density, *W*, is derived from classical Gaussian statistical thermodynamics, with a functional form, as in Equation (2), where *n* is the number of crosslinked chain segments per unit volume, *k* is the Boltzmann’s constant, and *T* is the absolute temperature.

(2) $$W=\frac{1}{2}nkT\left(I_{1}-3\right)$$

Note that Equation (2) is equivalent to Equation (1) with the elimination of the $C_{2}$ term and setting $C_{1}=nkT/2$. For simple geometries of rubber specimens, the neo-Hookean model can often be solved analytically due to its mathematical simplicity. Nonetheless, the model is inadequate for predicting stress–strain relations under high elongational stretch and under complex loading states such as in biaxial conditions.

An improved version of the Mooney model is the Mooney–Rivlin model [48,50], which extends Mooney’s model by incorporating a polynomial expression of the form ${\left(I_{1}\right)}^{i}{\left(I_{2}\right)}^{j}$, where *i* and *j* are non-negative integers. The Mooney–Rivlin model has been extensively utilized for several decades, although it has limitations in cases of biaxial loading with large stretches. Following similar reasoning, the Yeoh model [72] and the Gent model [73] proposed a power-like form and a logarithmic form, respectively. These two models have also been frequently used.

Given that for a general deformation gradient, F, the invariants, $I_{i}$, can be expressed as functions of the principal stretch ratios, $\lambda_{i}$, a class of hyperelastic models based on stretch-based continuum mechanics have also been developed. One example is the model proposed by Valanis and Landel [74], in which *W* is given by Equation (3), where the partial components, $w_{i}\left(\lambda_{i}\right)$, are experimentally obtained and do not contain adjustable parameters.

(3) $$W=\sum_{i=1}^{3}w_{i}\left(\lambda_{i}\right)$$

(4) $$W=\sum_{n=1}^{n}\frac{\mu_{n}}{\alpha_{n}}\left(\lambda_{1}^{\alpha_{n}}+\lambda_{2}^{\alpha_{n}}+\lambda_{3}^{\alpha_{n}}-3\right)$$

Following the stretch-based approach, Ogden [75] employed a compact form for *W*, as in Equation (4), where $\mu_{n}$ and $\alpha_{n}$ are constants needed to be calibrated by experimental data. The Ogden model can accurately capture stress–strain behavior of commonly used rubbers for nearly the entire stretch(es) range. Although the aforementioned phenomenological models can be of practical utility in fitting experimental stress–strain curves, they suffer from a few limitations, including (1) the parameter set optimized by fitting the material response to one type of deformation (e.g., uniaxial stretch) does not provide an accurate fit for the response to other deformation types (e.g., shear, biaxial stretch, etc.) for which a different parameter set needs to be developed, and (2) in most cases, the fitting parameters cannot be quantitatively linked with features of the network microstructure, and thus lack simple physical interpretability. To overcome such limitations, a full statistical description of chain-level behavior needs to be included. However, an exact mathematical expression for the free energy density, *W*, that incorporates a response in the non-Gaussian regime (as in Figure 5a) is difficult, and thus approximation is needed. Several approximation models have been introduced in the literature, including the 3-chain model [76], the 4-chain model [77], and the 8-chain model [51]. In the 3-chain model, three chains are aligned along each principal axis of a cubic unit cell, and stretches are applied to each chain affinely along the principal directions. The 3-chain model is effective in predicting uniaxial tension. However, it falls short in capturing the response to other types of deformation, such as biaxial tension, particularly in large stretch regimes. This is because the 3-chain approximation is unable to capture the strain-introduced anisotropy of the microstructure of the network. In the 4-chain model, an additional representative chain is employed to align with each vertex of a tetrahedron. Specifically, the four chains start from the center of the tetrahedron and end at the four vertices. The 4-chain model can partially account for large deformation behavior because the tetrahedron rotates when deformation is applied. The 8-chain model, as illustrated by Figure 5c, is based on the same essence as the 3-chain and 4-chain models. It assumes that the chains are diagonally connected to the vertices of a cubic cell. Due to the geometrical symmetry, the junction point located at the center of the cubic cell remains fixed under general deformation modes. This feature makes the 8-chain model one of the most widely used models to describe stress–strain behavior of rubber.

The chain-based models are based on the assumption of affineness, i.e., microscopic deformation of the network is identical to the macroscopic counterpart. The affine assumption was later refined in some models [71,78,79]. For example, Wu and van der Giessen advanced the affine model to a comprehensive framework to better account for complex localized deformations [79]. Still, these chain-based models are limited in the large deformation regime because nonaffine microstructure evolution under such deformations cannot be consistently treated in such models. For example, effects of entanglement (topological constraint) can only be captured by modifying the affine assumption [80]. An attempt to consider the nonaffine network behavior is the ABGI model [81], which adds an entanglement component and a tube model extension [82] to the 8-chain model. Additionally, the generalized hyperelastic microsphere model [70], as illustrated in Figure 5d, advances the features of the nonaffine network by considering two micro-state variables that are projected onto a microsphere to account for space orientations, i.e., a nonaffine stretch part and a nonaffine tube part. The model then defines a micro-to-macro transition based on a homogenized scheme. It was shown that the resulting 21-point scheme is sufficient to account for *W* of the network adequately, as shown in Figure 5e. This model, however, contains two micro-to-macro mean parameters, i.e., *p* and *q*, that do not have a direct physical interpretation and cannot be explicitly expressed in closed analytic forms. It is worth noting that some other models that explicitly consider the entanglement effects at the network level are available [52,80,83]. However, these models focus on the statistical treatment of the microscopic evolution of the chains and provide little guidance on the construction of the macroscopic free energy density, *W*. A few recent studies [53,84] have described new attempts to link the microscopic chain statistics to the macroscopic free energy density, *W*. These models attempt to incorporate the effect of entanglements into a crosslinked network while taking the nonaffine microscopic response of rubber materials into account. Following the model by Rubinstein and Panyukov [52], these studies employ the nonaffine tube model to represent the entangled network. In these models, the state of entanglement of a primitive chain with its neighboring chains is conceptualized as discrete anchor points on a continuous tube [68,69,85], which restricts the chain’s motion. In Figure 5f, the stress–strain curves predicted by the nonaffine model [53] are compared with experimental data.

In their simplest interpretations, both affine and nonaffine models appear to assume that the chains of a network contribute to the elasticity independently. However, in reality, the constituent chains may synergistically contribute to the elasticity. In such cases, assuming independent contribution may lead to an underestimation of the elasticity. For example, the effective contribution of a subset of chains can be modeled with assumptions of parallel or serial connections such that the chains contribute to the elasticity differently [86]. The parallel and serial arrangements represent topological features under two extreme cases of an ideal network. Topological representation of realistic networks is expected to fall between these two extremes when polydispersity (variation in chain length) and defects cannot be ignored [87,88]. To quantify complex topological features, improvement in measuring the local deformation of the chains is highly valuable. For example, Zhan et al. proposed a metric that evaluates the stretch of a chain by counting itself and its neighboring chains within a microsphere [89]. Although it was inspired by a preceding model by Bažant and Gambarova [90], the model by Zhan et al. applies to the loading cases of finite strains.

By construct, all constitutive models for hyperelasticity are fully reversible because the material properties, represented by the internal variables, are assumed to be independent of the applied strain rate. However, for real systems under dynamical loading, rate-dependent and dissipation processes cannot be neglected [91]. Additionally, the incorporation of filler particles within PMCs introduces reinforcement and inelastic effects, like the Mullins effect and the Payne effect, because of the evolution and interaction between the constituents. Therefore, corresponding constitutive descriptions must be modified to appropriately account for non-reversible processes, load-path dependence, and dissipation.

### 3.2. Reinforcement

The shapes of filler particles vary from sphere to aggregates, as mentioned, depending on the types of the particles. The fractal shapes of the aggregates have been supported by experimental observations [92,93]. The interaction between different particles can range from hydrodynamic-like to network-like, depending on the volume fraction, ϕ (or termed as loading) of the particles in the PMC and the interaction between the polymer matrix and the particles. Understanding the reinforcement mechanism is critical to guide the design for new PMCs, especially for those with NPs [94]. In the case of a small ϕ (∼0.05 or less) and well-disperse system, the inter-particle distance is much larger than the size of a chain-molecule (e.g., $\sqrt{\langle R^{2}\rangle }$), i.e., the particles are far away from each other, such that they particles behave independently. In such cases, reinforcement can be accounted for by the hydrodynamic effect because the particles interact with each other through the polymer matrix (particle–interface–bulk–interface–particle). There is no direct topological connection between the particles. As ϕ exceeds $\phi_{c,1}$, which is the critical condition (or threshold) for percolation, an infinite (i.e., macroscopically large) network of particles form within which every particle interacts with neighboring particle(s) via a layer of chains (particle–interface–particle). Microscopically, these neighboring particles are joined topologically by the polymer chains within the interface to form a percolated network, as shown in Figure 3, where the aggregate is joined by the bridging chains. When the inter-particle distance approaches zero as ϕ> increases to $\phi_{c,2}$ (the second critical condition), the particles form direct contact with each other such that particle–particle interactions contribute dominantly to the reinforcement and the inelastic properties of the polymer matrix composites [95], as illustrated by the agglomerates in the upper-left corner in Figure 3a. In this case, the particles form another type of network, which is energetically much stronger than the entropic network of the crosslinked polymer matrix or the percolated network of particles. We note that $\phi_{c,1}$ corresponds to the gel point of the percolation and is dependent on the conditions stated in Figure 1e.

#### 3.2.1. Hydrodynamic Effect

The hydrodynamic effect, first advanced by Einstein [96], is used to quantify the reinforcement when ϕ is small. In this theory, it is argued that the reinforcement is similar to the increase in solvent viscosity due to the introduction of spherical particles. The enhanced viscosity, η, of the mixture is given in Equation (5) [97], where $\eta_{0}$ is the viscosity of the pure solvent.

(5) $$\eta =\eta_{0}\left(1+2.5\phi \right)$$

However, the original theory only considers dilute mixtures with spherical additives. For higher ϕ, the increase in modulus is nonlinear [92]. The nonlinear effect is attributed to inter-particle interactions. One of the earliest nonlinear models was developed by Guth and collaborators [8,98], as given by Equation (6). To account for the effects of the non-spherical geometry of the filler particles, e.g., ellipsoidal shapes, the length aspect ratio, $r_{l}$, is included in Equation (7).

(6) $$\eta =\eta_{0}\left(1+2.5\phi +14.1\phi^{2}\right)$$

The Guth–Gold model (i.e., Equation (6)) has been widely applied to explain experimental observations for the reinforced modulus [92,99,100,101,102,103,104]. To obtain the modulus of the PMCs, the viscosity, η, is replaced by the shear modulus, *G*, or Young’s modulus, *E*, in Equations (5)–(7).

(7) $$\eta =\eta_{0}\left(1+0.67r_{l}\phi +1.62r_{l}^{2}\phi^{2}\right)$$

If $r_{l}$ is over ∼10, the particles can be considered as fibers such that classic theories for reinforcement composites may be adopted to quantify the reinforcement effect. The Halpin–Tsai model is also frequently used to fit the dependence of the modulus although it is usually considered empirical [105]. Another example is the Cox model [106] that considers aligned short-fibre additives. The Young’s modulus of the composite is given by Equation (8), where $E_{p}$ is the modulus for the filler. Here, *z* equals to $\frac{r_{l}}{2}{\left(\frac{2G_{0}}{E_{p}\ln\left(R/r\right)}\right)}^{1/2}$, where 2R is the separation distance of the filler and *r* is the radius of the filler. Interested readers are referred to specialized reviews [107,108].

(8) $$E=E_{0}\left(1-\phi \right)+E_{p}\phi \left(1-\tanh(z)/z\right)$$

#### 3.2.2. Percolated Network of the Filler Particles

Percolation and direct contact of particles enhance the stiffness of the PMCs by orders of magnitude [109,110]. According to percolation theory [111], an infinite cluster will be formed if $\phi >\phi_{c,1}$, although it is extremely difficult to precisely determine $\phi_{c,1}$ for a system of fractal-like filler-aggregate particles of different sizes. With the direct contact of particles, it is not surprising that most of the elastic energy will be stored in the contacted network of particles rather than the crosslinked polymer matrix. If the tenuous contact network is approximated by an ensemble of slender curved rods, the stored energy in the rods can be easily calculated [112] and thus the corresponding modulus of the contact network can be estimated [113].

The frequency-dependent complex shear modulus, $G^{*}\left(\omega \right)=G^{\prime }\left(\omega \right)+iG^{\prime \prime }\left(\omega \right)$, is also frequently used to characterize mechanical and viscoelastic properties of polymeric materials. For PMCs, $G^{*}\left(\omega \right)$ is assumed to be the sum of contributions from the polymer matrix and the percolated particles network, i.e., $G^{*}\left(\omega \right)=G_{matrix}^{*}\left(\omega \right)+G_{NP}^{*}\left(\omega \right)$. The first contribution, $G_{matrix}^{*}\left(\omega \right)$, is well-understood in the framework of hydrodynamic effects in the linear/nonlinear regimes, as discussed in the previous subsection. Additionally, one also needs to account for the presence of bound polymer layers adjacent to the particle surface, which results in an enhancement of the effective filler volume fraction, ϕ. This leads to modification of Equations (7) through (9), where $c={\left(1+2h/d\right)}^{2}$ includes the geometric effect of the interface into the effective volume fraction [114]. Here, *d* is the size of the particles and *h* is the thickness of the interface.

(9) $$G=G_{0}\left(1+2.5c\phi +14.1c^{2}\phi^{2}\right)$$

Although progress in quantitative understanding has been made, some open questions remain regarding the contribution of particle percolation. If the particles interact through the bridging chains, $G_{NP}^{*}∼{\left(i\omega \right)}^{2/3}$ according to critical percolation [66,115]. If the terminal relaxation modulus amplitude, $G_{NP}$, is known to be assisted by the structure of the particles, $G_{NP}^{*}\left(\omega \right)$ and thus $G^{*}\left(\omega \right)$ can be determined explicitly [109]. An order of magnitude increase in $G^{*}\left(\omega \right)$ can be accounted for by the collective motion of the percolation [116]. However, the model proposed by Chen et al. is limited to the case where separation between the filler particles is larger than the size, ∼b, of the chemical monomers but smaller than entanglement length, $bN_{ent}^{1/2}$, of the matrix polymer [109]. In the former case, the bridging chains that connect particles are in a glassy state rather than a rubbery state such that the particles interact via particle–interface–particle connection, causing the transition from percolation to contact network. In the latter case, the entanglement effect between the bridging and the matrix polymer chains competes with the percolation effect. This glassy-interface model only applies to cases where the applied strain is small or where the chains within the interface are unbreakable from the particle surface. On the other hand, the chains within the interface can be released or relaxed from the particle surface if the applied strain is finite or the interaction is relatively weak. Cui et al. showed that temperature-dependent desorption of chains is determined by interplay between the confinement level of the chains within the interface and the entanglement by a series of viscoelastic and infrared spectroscopy measurements [117]. The desorption and release of interfacial chains is usually used to account for inelastic bulk behavior of the composite materials, such as the Mullins effect and the Payne effect [118].

**Figure 6:**
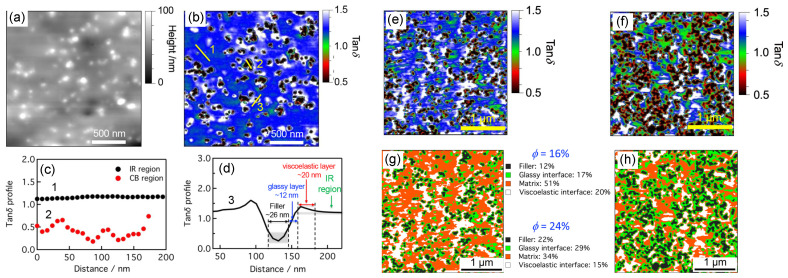
Viscoelastic profile probed by AM-AFM-based method that quantifies the reinforcement effect due to particle–interface–particle interaction. Adapted with permission from [125]. Copyright (2022) American Chemical Society. (**a**) The morphology and (**b**) the viscoelastic profile of SBR-CB PMC with ϕ=0.09. (**c**,**d**) The tanδ profiles for the matrices, particles, glassy, and viscoelastic layers. (**e**,**f**) The viscoelastic profiles of SBR-CB PMCs with ϕ=0.16 and ϕ=0.24, respectively. (**g**,**h**) The remapped profiles that distinguish different regions.

In the case of the particles being directly contacted, quantification of the modulus increase due to reinforcement is under active debate [119,120] because the increase is attributed to the fact that the interface is highly confined [121,122]. Such confinements lead to a significant change in the state of the interface from rubbery to glassy state with decreasing mobility [123], which enhances the stiffness of the interface and the PMCs [124]. In a recent experimental study [125], Nguyen and Nakajima used an amplitude-modulated atomic force microscopy-based method to directly probe the mechanical properties of different regions in the SBR-CB PMCs, especially for the samples with high ϕ. The $\tan\delta =G^{\prime \prime }/G^{\prime }$ was sampled across different regions such that the viscoelastic profile was obtained. In Figure 6a,b, the morphology and the viscoelastic profile of the SBR-CB PMC with ϕ=0.09 are shown. Different regions in Figure 6b are marked in Figure 6c,d, where the particles, interfaces, and matrices are differentiated with their sizes quantified. In Figure 6e,f, the viscoelastic profiles for the PMCs with ϕ=0.16 and ϕ=0.24 are shown, respectively. As ϕ increases, areas for the viscoelastic matrices (blue) shrink and those for the glassy interfaces (green and red) expand. The glassy regions merge as ϕ increases. The viscoelastic profiles can be further decomposed into distinct components, as shown in Figure 6g,h, for PMCs with ϕ=0.16 and ϕ=0.24, respectively. The glassy (green) regions in Figure 6g are mostly isolated such that the nonlinear reinforcement is highly attributed to percolation of the filler particles. However, clusters of particles connected by the glassy interface in Figure 6h are prominent so that contact network dominates the nonlinear reinforcement. It is worth noting that the decomposition is based on the Gaussian mode-fitting of the viscoelastic profiles such that the results are biased by predefined values of tanδ. Interestingly, in the cases of the particles being directly contacted or agglomeration of the particles, occluded volumes will be created if neighboring clusters of particles enclose topologically, as shown in Figure 3b. In such a case, the occluded volume can be in a complete glassy state, without any contact with the rubbery matrix. Although the occluded chains are shielded from the stress-bearing matrix, they increase the effective volume fraction, ϕ, of the filler particles so that the level of reinforcement can be even higher than the bound layer [126].

### 3.3. Viscoelasticity

**Figure 7:**
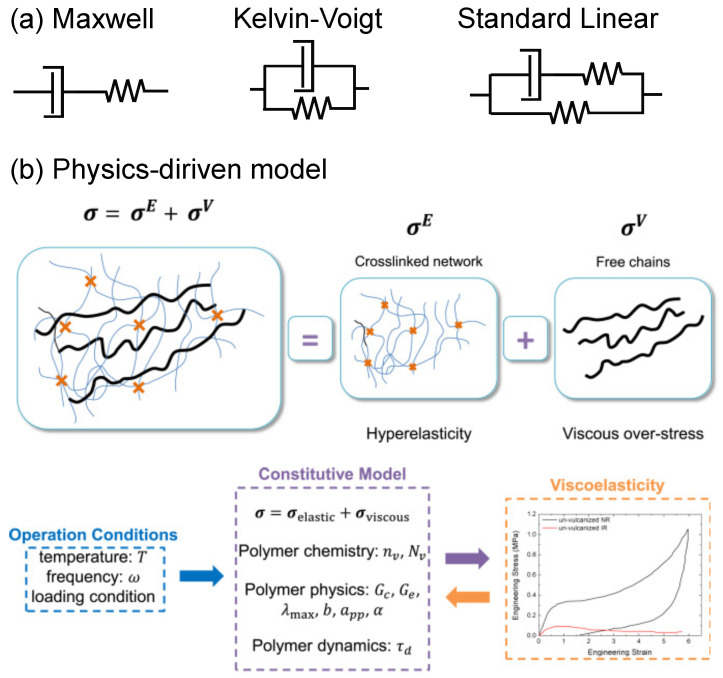
Viscoelastic models. (**a**) Three classical viscoelastic material models, including the Maxwell, Kelvin–Voigt, and standard linear solid (SLS) models. (**b**) Example of a physics-driven model that explicitly includes the relaxation spectrum of polymer molecules to establish the macroscopic viscoelastic response. The orange crosses represent crosslinks that connect network chains. Adapted from [62] with permission from Elsevier Copyright (2016).

Another characteristic property of elastomers is the viscoelastic behavior under rate-sensitive loading when the strain rate is comparable to the intrinsic relaxation time, τ, of the material system. Understanding this behavior involves analyzing the time-dependent microscopic evolution of the material. For unentangled free chains, the Rouse and the Zimm models are well developed, while for entangled systems the tube model, along with conceptual extensions like chain-contour-length fluctuations and constraint-release mechanisms, is widely recognized [127]. When the chains are crosslinked, diffusion is suppressed and chain dynamics confined by the crosslinking sites [91]. Early attempts to model viscoelasticity were phenomenological in nature and employed mechanical analogy with different arrangements of springs and dashpots. More popular among these models include the Maxwell, Kelvin–Voigt, Burger, and standard linear solid (SLS) models, as shown in Figure 7a. However, simple models with spring/dashpot elements were found to be insufficient to capture the wide spectrum of relaxation time(s) τ observed in reality. A logical improvement is to use a combination of multiple viscoelastic elements, as in the SLS model. There have been other approaches for building models that capture relaxation behavior over a wide time range [128]. A classic example is a model in which the relaxation (or creep) function, also known as the memory kernel, is expressed as an additive combination of exponentials with different characteristic time constants, known as the Prony series. Combinations of various phenomenological models can be used to develop a generalized constitutive model [129]. Additionally, linear viscoelasticity (LVE) theories make use of the Boltzmann superposition principle, which makes it possible to express the dynamical response of polymeric systems to different loading conditions in terms of simple convolutional integrals. Such formulations are amenable to easy numerical implementation with finite element codes. Other memory kernels have also been used in the literature, including the rational polynomial form or fractional power function [130,131,132]. Such models require high-quality experimental data to calibrate many of their parameters. Viscoelastic models based on fractional calculus require fewer parameters but necessitate experimental observations that exhibit approximate power-law behaviors [133,134]. The major problem of fractional viscoelasticity is its high computational cost due to the difficulty in numerical implementation, and the parameters of the fractional kernel often lack satisfactory physical interpretations [135].

LVE models are appropriate for describing viscoelastic response under small external stimuli. Nonlinear viscoelastic models, on the other hand, have been formulated to describe the time-dependent response of rubber-like polymers and bio-related tissues when subjected to finite/large deformations. To date, many models have been developed to quantitatively describe finite strain viscoelasticity observed experimentally. Several early models use only phenomenological arguments to account for the viscoelastic stress–strain behavior [136,137,138]. These models can be categorized into two types depending on whether they incorporate tensorial stress-like or strain-like internal variables, allowing their generalization to three-dimensional cases. Some of these works [55,60,139] adopt vectorial internal stress variables for nonlinear viscoelasticity. In contrast, other models [140,141,142] are formulated on finite-strain theory in which the total deformation is decomposed into reversible and irreversible parts, where the irreversible deformation is represented by strain-like internal variables. Inspired by the classic Rouse and tube models, there have also been attempts to develop physics-driven viscoelasticity models. For example, the viscoelastic response of different polymeric melts has been shown to be well captured by a constitutive model based on a multiscale predictive framework that explicitly includes the Rouse and the reptation dynamics [143].

Other nonlinear viscoelastic models incorporate microstructural mechanisms for free chain motions in characterizing the viscoelastic behaviors of hyperelastic solids. Bergstrom and Boyce [144] pioneered work in continuum modeling of nonlinear viscoelasticity. In their work, they conceptualized the mechanical behavior of viscoelastic materials as a combination of an elastic part and a viscous part. The elastic part is viewed as driven by the state of equilibrium that can be attained over long-term relaxation, while the viscosity is attributed to the nonlinear deviation from this equilibrium state. Miehe and Göktepe [61] followed a similar decomposition strategy and formulated the viscous part in terms of two micro-kinematic mechanisms, which involve the stretch and area contraction of the tubes. Although models such as these attempted to describe the nonlinear viscoelastic behavior of polymers through insightful microstructural mechanisms, they still necessitated inclusion of a few phenomenological parameters. This made predicting polymers’ macroscopic responses from a bottom-up approach challenging. To address this issue, Tang et al. introduced a two-scale, physics-driven theory for nonlinear viscoelastic elastomers that included the effect of intrinsic relaxation of polymer chains [62,145], as shown in Figure 7b. Their method employs a multiscale framework, which enables the prediction of macroscopic viscoelastic properties through a series of bridging laws that span from the micro to the macro scale. The approach not only elucidates the nonlinear behavior of rubber elasticity but also analyzes the microstructural evolution of free chain motions. In this framework, the tube survivability function [146,147], a relaxation concept developed within the reptation model [69], is used as input to the viscoelastic memory kernel. In addition, the standard Mittag-Leffler function is used to account for the fractional diffusion of the free chains. The resulting survivability function is given in Equation (10), where *s* is the segment index (contour length variable) along the primitive chain that ranges from 0 to *L*. Here, *L* is the total contour length of the chain. *E* is the Mittag–Leffler expression, with α the fractional index. The tube survivability function, Ψ(s,t), represents the relaxation spectrum of the free chains by accounting for the possibility that chain segment *s* is still inside the original tube at time *t*. This relaxation spectrum is then translated to the spatial orientation of the free chains such that the viscoelastic stress is calculated accordingly. A schematic of the framework is shown in Figure 7b. With the incorporation of filler particles, the viscoelastic behavior of the PMC can be significantly changed. For example, volume fraction of filler particles is related to change of the temperature-dependent viscoelastic response of the PMC such that the volume fraction can be used to tune the material response to obtain the desired level of energy dissipation [148].

(10) $$\Psi \left(s,t\right)=\sum_{n=1,odd}^{\infty }\frac{4}{n\pi }\sin\frac{n\pi s}{L}E_{\alpha ,1}\left(-{\left(\frac{n^{2}t}{\tau_{d}}\right)}^{\alpha }\right)$$

### 3.4. The Mullins Effect

**Figure 8:**
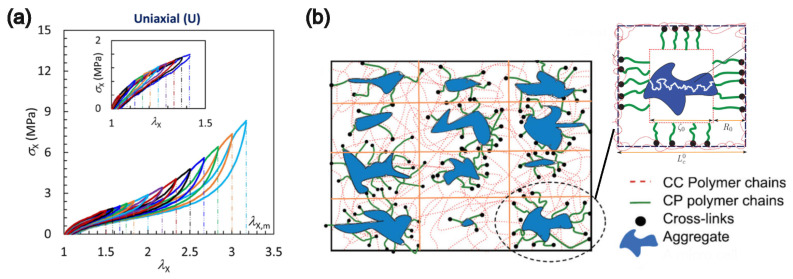
The Mullins effect. (**a**) Stress–strain curves showing the Mullins effect. Loading cycles are represented by different colors. Reprinted with permission from [149]. Copyright (2022) American Chemical Society. (**b**) Schematic of network decomposition into CC, PP, and CP sets of chains that correspond to hyperelastic, history-dependent damage and viscoelastic dissipation, respectively. Reprinted figure with permission from [56]. Copyright (2013) by the American Physical Society.

The Mullins effect describes a substantial decrease in stiffness of filled elastomers or even their unfilled counterparts under loading/unloading cycles. The decreases in stiffness persist in the subsequent loading cycles unless the applied level of deformation exceeds previously reached maximum levels, as shown in Figure 8a for carbon-black-filled-rubber SBR-CB [149]. The Mullins effect is also associated with hysteretic energy dissipation during the cyclic loading. The first observation of the Mullins effect dates back to the early era of the rubber industry [150] although the phenomenon was not systematically studied until a detailed set of experiments by Mullins and his colleagues [54,151,152,153]. The effect is more pronounced in filled elastomeric systems and has been observed across different deformation modes [154,155,156,157]. The decrease in stiffness and the hysteresis are attributed to microscopic damage in the material, especially involving irreversible changes within the matrix–particle interface. Theoretical treatments of such damage evolution have been developed based on methods ranging from phenomenological to physics-driven modeling. The Mullins effect can be quantitatively described by the softening, permanent set, and the hysteretic energy loss. Decrease in the elastic modulus of the material is a direct observable related to the softening, as slopes of the unloading curves are smaller than those of the loading curves in Figure 8a. Permanent sets are the residual deformations that the materials remain in without applied stress. Hysteretic energy loss is calculated by the area enveloped by a loading and its subsequent unloading stress–strain curves.

In the simplest models, the matrix–particle composite is represented as a binary composition of independent hard and soft components [54]. Pure geometric consideration is then used to derive a stress–strain response relation for the composite. Loading-history dependence is incorporated through the introduction of a damage function, usually to the hard segment, which depends on the loading history, more specifically on the previously reached maximum stretch levels [158]. Such a phenomenological damage function approach has been widely adopted because of its intuitive appeal [159,160]. A number of continuum models in the literature have been motivated by various microscopic damage mechanisms. Simo formulated a rate-dependent isotropic damage model and applied it to fully three-dimensional finite-strain simulations [55]. With a slightly different functional form for the isotropic damage evolution, the quasi-static model by Ogden et al. captures the Mullins effect in multiaxial loading states [155]. In addition, a second internal variable was later introduced to quantify the residual strain [161]. Besdo and Ihlemann borrowed the idea of Tresca’s yield criterion to formulate the damage function such that the influence of geometric nonlinearity on the damage is accounted for during finite deformation under different loading conditions [162]. Another classic treatment is the strain amplification concept under the hypothesis that the stress–strain response can be quantified by an equivalent amplified strain for the soft component [54]. This treatment has been widely applied to isotropic and anisotropic cases [163,164].

Although the phenomenological damage models discussed above provide a practical framework for computing the loading-history-dependent hysteretic response, such models likely involve an oversimplified representation of underlying microscopic processes in real materials. Insights into complex dissipation mechanisms responsible for the Mullins effect have been slowly emerging since the pioneering works of Mullins and later researchers. For the unfilled matrix, the softening effect is attributed to the breakage of chemical crosslinks, as well as the rupture of and residual local alignment of the network chains [165]. In the case of a filled system, the Mullins effect is understood to occur due to the tearing of polymer chains from and between filler surfaces [166,167]. In addition, reversible processes like sliding of the polymer chains along the filler surface are also important [168]. Additional processes involve localized damage of the filler agglomerate structure, as supported by experiments on filled SBR [169]. Therefore, it is not surprising that phenomenological treatments that condense such a variety of inelastic mechanisms into limited parameters probably obscure the connection between microscopic structural changes and the macroscopic mechanical behavior of the polymer matrix composites.

Over the years, there also have been attempts to develop and refine constitutive models based on some of the microscopic mechanisms discussed above. Given the importance of describing chain breakage and chain sliding processes [165,170], it was natural for these models to distinguish the polymer chains within the bulk matrix from the chains in the vicinity of the filler particles, in essence defining two classes of chains, i.e., network chains and particle chains, as shown in Figure 8. The initial model, developed by Simo and Govindjee, involved a three-chain representation of the system [171]. Göktepe and Miehe expanded on such ideas, with the first and second types of chains being termed CC and PP sets, with an implementation involving a more elaborate 21-point scheme [61]. The decomposition of the polymer chains into matrix and particle subsets enables meaningful physical definitions of the model parameters. The free energy functional forms for both $W_{PP}$ and $W_{CC}$ components were formulated using previously established theories of hyperelasticity. Additionally, damage evolution of $W_{PP}$ needed to be modeled. While phenomenological differential equations, like those in the literature [55,155], are legitimate choices, a more sophisticated way is to base the damage evolution on the chain-length distribution of the PP set because the distribution essentially changes with the damage. For example, Dargazany and Itskov proposed that the chain length of the PP set follows a statistical distribution rather than being monodisperse, and the effect of deformation is to alter this distribution in ways that depend on strain history [118]. It is assumed that relaxations of the chains are much faster than the applied deformation rates such that the chains are always in equilibrium. There is no phenomenological damage parameter included in the free energy $W_{PP}$. Another important aspect of this model was to incorporate effects of single-chain free energy into $W_{CC}$ and $W_{PP}$ of the continuum. The 21-point integration scheme [61] was found to be more robust than the 3-chain [171] and the 8-chain schemes [51], as it captures the strain-induced anisotropic distribution of not only the CC chains, as previously mentioned in the discussion on hyperelasticity, but also of the PP chains for the damage evolution [118]. A comparison between the 21-point and the 8-chain schemes is summarized by Diani et al. [172]. Additional chain sets beyond the CC and PP sets have also been included to achieve greater flexibility in quantifying inelastic phenomena of the PMCs. This is because the CC and PP sets are topologically isolated and thus independent from each other. Such an assumption excludes the chain set, named CP, that bridges the CC and the PP sets. When the interaction between the CC and the PP sets cannot be ignored, it is important to include the free energy contribution, $W_{CP}$, from this third set. In their follow-up work, Dargazany and Itskov included the microscopic yield behavior of filler agglomerates such that the macroscopic hysteresis of the PMC can be quantitatively understood [56]. The free energy density of the PMC is given by $W=W_{CC}+W_{PP}+W_{CP}$. The network decomposition is illustrated in Figure 8b. The decomposition enables separation of stress contributions from different networks, as in Figure 8b, where CP chains and the aggregate of particles lead to the viscoelastic energy dissipation while the PP components represent the damage-induced energy dissipation. While $W_{CC}$ is time-independent, $W_{PP}$ and $W_{CP}$ are time-dependent, such that $W_{PP}=W_{PP}\left(t,\lambda_{max}\right)$, where $\lambda_{max}$ is the maximum stretch level experienced by the material so far.

### 3.5. Strain-Induced Anisotropy

There are additional factors that can lead to strain-induced anisotropy. One example is strain-induced crystallization (SIC), a phenomenon that is widely observed (even without the fillers) [173]. There have been many contributions to the continuum models of SIC since the seminal work by Flory [174]. Because of the inherent molecular structure of polymer chains, the crystallization behavior of polymer materials is generally different from and more complex than that of metals, which have well-defined lattice structures [175]. Polymer chains interfere with themselves during the alignment process of crystallization. This leads to an incomplete crystallization process and results in a semi-crystalline material in which both amorphous and crystallized phases co-exist, with the possibility of transformation from one phase to the other. It is important to capture such a multiphase nature of the semi-crystalline polymer in a material model. To quantify the stress–strain behavior of semi-crystalline polyamide, Regrain et al. implemented a phenomenological model based on a framework of infinitesimal strain plasticity in which the amorphous and the crystalline phases acted independently from each other [176]. Ayoub et al. incorporated multiple viscoplastic dissipation mechanisms to describe the transition between the amorphous and crystalline phase [177,178]. The first mechanism is rate-dependent and is modeled by an activation-like process described by the Arrhenius law [179]. A second mechanism is via strain-induced alignment of the crosslinked chains. While the model can quantify strain hardening during the loading process, it is inadequate in describing the unloading behavior. This is because the model relies on a yield argument that assumes irreversibility of the strain-induced alignment. Another limitation of the model arises out of the isotropic formulation of the flow rule, which goes against the anisotropic nature of the multiphase materials. More specially, the isotropic formulation prohibits tracking the strain-induced anisotropic evolution of the microstructure. One way to introduce anisotropy is to assume that many crystallographic systems are simultaneously active at a material point [47], with anisotropy being governed by predefined crystallographic systems [180]. Another way to include anisotropy is to construct the free energy density, *W*, within a representative volume element (RVE) using micromechanics arguments. By assigning different phases to the active material points, the microscopic anisotropy caused by phase distribution can be captured mathematically. For example, Popa et al. [181] proposed a model based on a polyhedral volume RVE with 20 corners and 27 Gauss points that specify the phase distribution. Homogenization over the RVE enables the estimation of *W* macroscopically. One of the advantages of the introduction of anisotropy is the possibility to track the structural evolution-like texture formation [182], which may influence the mechanical properties of the materials significantly.

As discussed above, to model the semi-crystalline polymer system it is important to not only describe the crystalline and amorphous phase distribution of the microstructure quantitatively, but also the transition between the two phases. As an example, the anisotropic model implicitly includes such transition through the kinetic constraint that the total volume of different phases remains constant [180]. However, such a constraint can depend too sensitively on the boundary value problem and may not capture the intrinsic transition law of the material. Concerning the chain set decomposition, semi-crystalline phases can be assigned as fibrillar ST and lamellar FL sets according to their morphologies [183,184]. The fibrillar crystal is formed by different aligned chains along a shared direction while the lamellar crystal is formed by a single chain folding back and forth. In this framework of decomposition, the ST and FL sets interact only with the PP set, and the degree of crystallization is controlled by a single internal variable that evolves during the applied deformation. A simple natural choice of such an internal variable is the fraction of the crystal phase including the ST and the FL sets. However, the ST and the FL may interact with the CC set such that additional internal variables are necessary to model these interactions. Discussion of an exhaustive list of works focusing on the continuum modeling of semi-crystalline polymers is outside the scope of this review. Interested readers are suggested to refer to specified reviews [185,186]. Nonetheless, it remains a challenge to link microscopic crystallization processes to the macroscopic mechanical properties in bulk under the co-existence of different phases. While microscopic crystallization can be experimentally probed by scattering [187,188,189], the modeling of interactions between the different phases at the continuum level still relies on certain geometric assumptions [190].

Introducing particles into the semi-crystalline matrices significantly alters the kinetics and structures of the materials. For example, Corté and Leible showed that the toughness of semi-crystalline polyamides with dispersed NPs is strongly related to the inter-particle distance [191]. The toughness increases as the inter-particle distance decreases because of the glassy polymer matrix argument presented in the discussion of the reinforcement effect. In the case of large filler particles where particle diffusion is much slower than the matrix polymer chains, the particles have two roles: act as the heterogeneous nucleation sites and provide spatial confinement to the crystallization processes [192].

### 3.6. The Payne Effect

**Figure 9:**
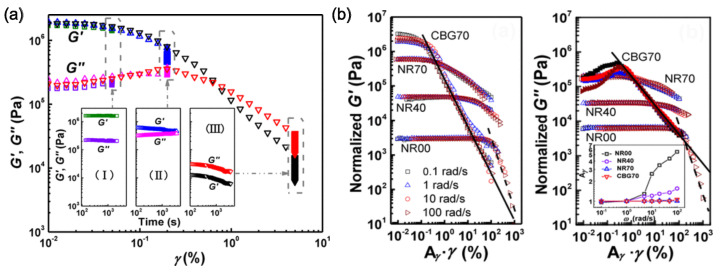
The Payne effect is demonstrated by the complex modulus, $G^{*}$, as a function of the strain amplitude, γ. Adapted with permission from [196]. Copyright (2016) American Chemical Society. (**a**) Non-monotonic behavior of the loss modulus, $G^{\prime \prime }$. (**b**) Payne effect is significant if the loading of particle ϕ is high.

The discussion of the Mullins effect in the PMCs in the previous section assumes a loading history that is static or quasi-static. However, the mechanical response properties of the composites to frequency-dependent loading conditions are also of great importance because the PMCs are frequently used as damping components like bushings and couplers in automobiles. Frequency-dependent properties of CB-filled PS and PB, which were extensively studied in the 1940s and 1950s, displayed interesting behavior as a function of deformation amplitude and filler content. Such behavior subsequently came to be known as the Payne effect [57,193,194,195]. The main effect is the observation that, under cyclic loading conditions, the storage modulus, $G^{\prime }$, decreases when the amplitude of shear strain exceeds a certain level and the loss modulus, $G^{\prime \prime }$, shows a non-monotonic effect, as illustrated in Figure 9a, where results are from experimental measurement by Gan et al. [196]. In addition, the Payne effect also describes the complex modules, $G^{*}$, influenced by the loading of particles, ϕ, as in Figure 9b. The $G^{\prime \prime }$ of the pure NR and NR40 (moderate ϕ) remain constant until the shear thinning regime where the moduli decrease with the increased amplitude of the applied strain. However, NR70 (high ϕ) and CBG (the percolated network of the filler CB) rubbers show a significant Payne effect, i.e., softening happens at much lower strain amplitudes.

Being a frequency-dependent phenomenon, the Payne effect can be generalized by viscoelastic arguments such that the free energy density, *W*, is derivable once the relaxation spectrum, $G\left(t\right)$, is available. Overall, the microscopic mechanism behind the effect is believed to be that under two cyclical load processes, i.e., the breakage and reformation of weak bonds, especially within the inter-particle interface, which compete with each other. It is their net effect as a function of the strain amplitude that determines the macroscopic equilibrium. An increase in the strain amplitude accelerates the breakage of the weak bond while slowing the reformation. One of the classic models of the shear-amplitude dependence of the complex modulus, $G^{*}$, was proposed by Kraus [58], as in Equations (11) and (12), where $G_{0}^{\prime }$ is the storage modulus at very small strain levels (<0.01%), $G_{\infty }^{\prime }$ and $G_{\infty }^{\prime \prime }$ are asymptotic values of the storage and loss modulus at very large strain levels, and $G_{m}^{\prime \prime }$ is the maximum loss modulus, which occurs at a strain amplitude level of $\Delta \gamma_{c}$. A collection of models based on the Kraus model has been developed in the subsequent literature [95,197,198,199,200].

(11) $$G^{\prime }\left(\Delta \gamma \right)=G_{\infty }^{\prime }+\frac{G_{0}^{\prime }-G_{\infty }^{\prime }}{1+{\left(\Delta \gamma /\Delta \gamma_{c}\right)}^{2m}}$$

(12) $$G^{\prime \prime }\left(\Delta \gamma \right)=G_{\infty }^{\prime \prime }+\frac{2\left(G_{m}^{\prime }-G_{\infty }^{\prime }\right){\left(\Delta \gamma /\Delta \gamma_{c}\right)}^{m}}{1+{\left(\Delta \gamma /\Delta \gamma_{c}\right)}^{2m}}$$

The Kraus model is purely phenomenological and does not provide any physics-based connection between the microscopic structural evolution and the complex modulus, $G^{*}$. A more physically motivated theory was later proposed as the van de Walle–Tricot–Gerspacher (VTG) model [201]. In this model, the Payne effect is attributed to linear viscoelastic behavior of the polymer matrix and nonlinear response resulting from inter-particle interaction, respectively. While the polymer matrix is modeled as a Maxwell material, the overall inter-particle interaction is modeled as a competition between the viscous drag from the matrix and the intrinsic Lennard–Jones potential between particle pairs, with three- and higher-order-body interactions ignored. A threshold strain amplitude, $\varepsilon_{0}$, is defined such that the nonlinear inter-particle contacts are switched on and off in a stepwise manner. The complex modulus, $G^{*}$, of the PMC is consequently determined by the counterpart, $G_{\infty }^{*}$, of the matrix, a constant *h* related to the viscoelastic energy dissipation, and the weight function, $N\left(\varepsilon ,\varepsilon_{0}\right)$, that controls the non-monotonic behavior of $G^{\prime \prime }$. However, calibration of these material-dependent parameters relies on experimental data, and the individual parameters are not amenable to simple physical interpretations.

For an improved description of inter-particle interaction, properties of the filler particles must be included in the $W_{PP}$ formulation. The network junction (NJ) model is similar to the VTG model but explicitly considers the volume density of contact points and the specific surface area, *S*, which allows $G^{*}$ of the PMC to be quantitatively expressed as a function of the filler volume fraction, ϕ, and the viscosity, η, of the polymer matrix [114]. However, since the NJ model was derived based on static conditions, it is limited in predicting the non-monotonic behavior of the Payne effect. The links–nodes–blobs (LNB) model assumes a contact network of the filler aggregates, which in a simplified form is represented as an energetic network [202]. While the blobs and the links are, respectively, formed by tightly and loosely connected filler aggregates, a node is defined as a location where a link splits. Mechanical properties of the energetic network are controlled by the tensile and bending stiffness of the links $G_{link}$ and $Q_{link}$, respectively, which are used to express the free energy of the energetic network as a function of the applied strain. The LNB model is an oversimplified representation when the energetic network is of fractal nature. A more sophisticated description of the energetic network is given by the cluster–cluster aggregation (CCA) model [59,113]. In this model, the volume fraction of the filler is given according to Flory’s fractal dimension, $d_{f}$, for the aggregation of the primary particles, as $\phi \left(\xi \right)={\left(d/\xi \right)}^{3-d_{f}}$, where *d* is the primary particle size and ξ the aggregate size. The corresponding modulus of the PMC is given by Equation (13), where $d_{f,B}$ is the fractal number for the arms of the network formed by the aggregates and *h* is the thickness of the interface. $G_{p}$ is the modulus of the aggregate network, as derived in the LNB model. Equation (13) represents a power law for *G* as a function of ϕ, and it was shown that $G∼\phi^{3.5}$ [113,203]. Equation (13) also shows that *G* increases nonlinearly as a function to *d*. It is worth noting that, because of the energetic nature, the aggregate networks in the LNB and the CCA models are conceptually identical to the contact network presented in Section 3.2.2. However, in the case of the percolation network of aggregates, the bridging chains that connect the filler aggregates contribute entropically to the free energy, $W_{PP}$, while the interface of adsorbed chains on the aggregate surface behaves energetically. Therefore, the applicability of the LNB and the CCA models to describe the percolation network is somewhat debatable.

(13) $$G=G_{p}{\frac{{\left(d+2h\right)}^{3}-6dh^{2}}{d^{3}}\phi }^{\frac{3+d_{f},B}{3-d_{f}}}$$

Equation (13) provides a way to describe how the strain-dependent modulus, $G^{*}$, changes with the structural change of the aggregate network. In small strain cases, the elasticity of the PMC is dominated by the almost rigid filler aggregate network, since this network is much stiffer than the polymer matrix. The deformation is essentially elastic with little dissipation. As the strain amplitude increases, the modulus of the PMC starts to decrease due to progressive weakening of the network through larger deformation. Additionally, the deformation of the network dissipates energy due to friction between the aggregates, which is marked by an increase in the loss modulus, $G^{\prime \prime }$. With a further increase in strain amplitude, the arms of the network are broken into isolated aggregates. The structural integrity of the network deteriorates until reinforcement is dominated again by hydrodynamic effects. The phenomenon can be quantitatively described in terms of the number of surviving aggregate clusters in the network. The resulting storage modulus, $G^{\prime }$, is given by Equation (14), where m∼0.5 is another fractal number and τ∼3.6 is universal to the percolation aggregate network regardless of the filler types [112]. The CCA model has been successful in interpolating experimental results [204,205].

(14) $$G^{\prime }\left(\gamma \right)=G_{p}^{\prime }{\left[1+{\left(\frac{\gamma }{\gamma_{c}}\right)}^{2m}\right]}^{-\tau }$$

## 4. Smart Materials

Although historically the use of fillers in polymer matrices has primarily been for reinforcement purposes, more recent efforts have also been exploring other functionalities with specially designed particles. In this section, three types of filled materials including magnetoelastic, shape-memory polymer (SMP), and self-healing polymer (SHP) composites are reviewed. It is worth noting that there are more types of smart materials than what are covered in this review. For example, dielectric elastomer is another smart material frequently used as artificial muscles [206] to achieve controllable motions for applications like swimming strokes [207]. But we focus on the first three types in this work. The mechanistic and physical views regarding the constitutive models are presented with discussions of some material details and possible applications.

### 4.1. Magnetoelastic Materials

Magnetoelastic PMC (M-PMC) is an emerging material that has drawn great attention in recent years because of its potential in robotic, biomedical, and industrial applications [42]. The magnetic response of such materials originates from embedded particles. Activation and motions of M-PMC are usually driven by external magnetic fields, enabling contactless setup and therefore flexible utilization and control strategy. M-PMCs have particles with sizes ranging from a few nanometers to hundreds of micrometers, such that their surface-to-volume ratio is sufficiently high. Mechanical properties of M-PMC are influenced by intrinsic properties, shapes, and spatial distributions of the embedded magnetic particles. Magnetic particles can be categorized by their magnetic characteristics into hard-, soft-, and superparamagnetic types, which can be distinguished by the magnetization (B−H) relation that records the relation between the magnetic field (*B*) within the material and the applied stimulus field (*H*). Hard-magnetic materials show large hysteresis in the B−H curves such that their magnetic remanence and coercivity are high. Magnetic remanence ($B_{r}$) quantifies residual magnetization of the material when *H* vanishes, while magnetic coercivity ($H_{c}$) is the amplitude of *H* required to demagnetize the material. Soft-magnetic materials, however, have much smaller $B_{r}$ and $H_{c}$, compared with hard-magnetic counterparts, so the hysteresis in the B−H curve is low. In the extreme case of very fine particles, the B−H hysteresis almost vanishes, and the magnetization can be easily quenched by thermal fluctuations. Hard-magnetic and superparamagnetic particles are more commonly used in M-PMCs for actuation applications. This is because they both can maintain high magnetization levels, the former without an external field and the latter with an external field, respectively. In contrast, soft-magnetic particles support low magnetization levels, even under high external fields. Therefore, M-PMCs based on soft-magnetic particles are less commonly used in practical applications.

The shapes of the magnetic particles are also important to their functionality. If spherical particles with randomly oriented magnetic axes are uniformly embedded in the M-PMC, the external field-induced deformation response of the M-PMC will be directional extension or contraction because the magnetization direction of each particle tends to align with the external magnetic field. When the particles are non-spherical with a high aspect ratio (e.g., rod-like and ellipsoidal), bending deformation can be achieved as a result of torque generated by the rotation of the particles under an external field [208]. While hyperelastic polymer networks like PDMS elastomer, polyacrylamide, and poly(vinyl alcohol) gels are commonly used in biomedical applications because of their excellent bio-compatibility and low toxicity, recent works have explored the use of a responsive polymer matrix to achieve synergistic effects between the active matrix and the magnetic components. For example, SMPs and liquid-crystal elastomers (LCEs) are promising choices because their transitions between states (as discussed later) can be harnessed, together with the magnetic component, to achieve complex driving motions. The strategy to design an appropriate material and structure to accomplish a desired motion mode can vary according to the application. The most straightforward approach is to design the structure of the M-PMC and pattern the distribution of the magnetic particles without any additional activation mechanism [209,210]. For example, Jiralerspong et al. designed kirigami patterns for the M-PMC made of iron oxide NPs and a silicone matrix [211]. Two-dimensional in-plane and three-dimensional out-of-plane activated motions were achieved by patterning the direction of magnetization of the magnetic particles in individual units of the kirigami.

By direct printing or injection molding, M-PMC structures are fabricated to accomplish flexible locomotion, which has great potential for applications in healthcare [212,213,214,215,216]. Such a strategy can facilitate high-throughput and low-cost manufacture of soft machines and robotics when combined with magnetic reprogramming strategies that reorient the magnetic direction of the particles, which leads to a controlled redefinition of the deformation pattern [217]. M-PMCs with diverse deformation modes and other properties have been developed using SMPs and LCEs with or without combining them with an inert matrix. Integration of magnetically passive elastomers and responsive SMPs, especially by direct ink-writing techniques, can yield structures that are capable of morphing under both thermal and magnetic stimulus [218,219]. The properties of SMP-hybrid M-PMCs can be further manipulated by substituting permanently crosslinked matrices with chemically flexible systems that can change their atomistic structures through the thermally reversible Diels–Alder reaction [220]. As a result, not only the magnetic characteristics but also the physical shape of the M-PMCs can be reprogrammed to meet specific design requirements. On the other hand, LCE-hybrid M-PMCs are preferable in some applications because the morphing and motion of the LCE matrix can be triggered by light [221]. Another advantage of LCE-hybrid M-PMCs is that they can sustain more cyclic loading than their SMP counterparts because the state transitions of LCEs are usually reversible [222]. Nonetheless, sequential responses can be embedded into both LCE-hybrid and SMP-hybrid M-PMCs that can adapt their properties according to the service environments [223,224,225].

It is worth noting that some of the generic structural properties of PMCs apply to M-PMCs as well. For example, the distribution of the magnetic particles within the matrix can change from uniformly dispersed to aggregate/agglomerate topology such that the responsive morphing behavior of M-PMCs changes, often uncontrollably, as a function of time, loading cycles, and ambient conditions. However, one can design clever strategies to overcome any unwanted degradation in responsive performance and at the same time tune the mechanical properties of the M-PMCs. For example, the interfacial interaction between the magnetic particles and the polymer matrix is important in controlling many of the properties of the M-PMCs. If the binding of the interface degrades due to particle agglomeration, the magnetic response of the particles is not transformed efficiently to the mechanical power that drives the morphing motion of the M-PMCs. One could address this problem by coating the particles with appropriate chemical groups so as to improve the interfacial interaction [214,226,227].

**Figure 10:**
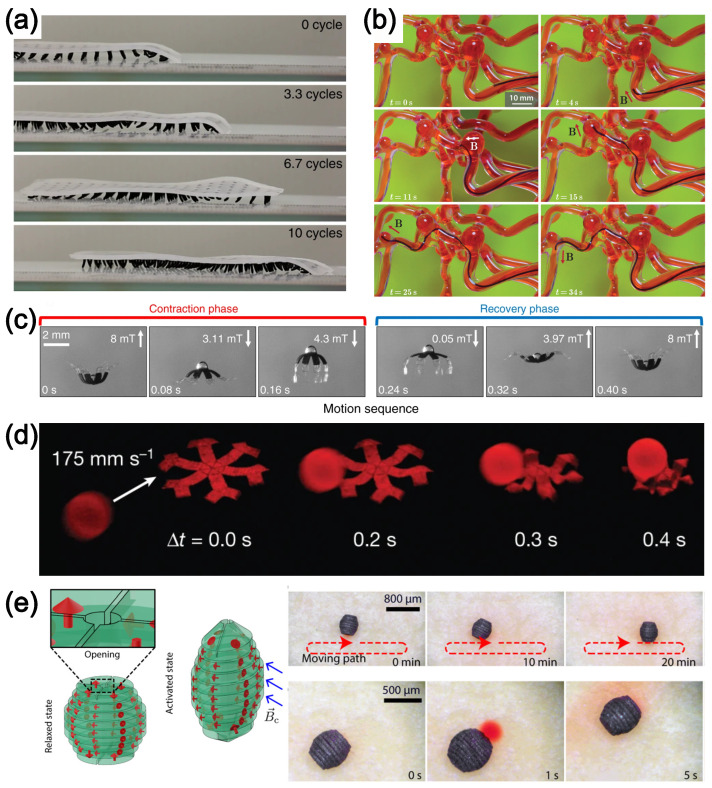

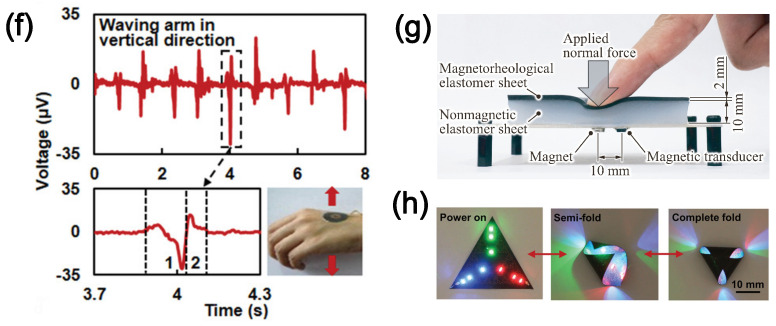
Application of the M-PMCs. (**a**) Locomotion of the soft substrate (opaque) structured with magnetic composite pillars (black) is driven by a metachronal magnetic field. Adapted from [228]. (**b**) Manipulation of a magnetic responsive thread in the model 3D cerebrovascular space. Adapted with permission from [214]. Copyright (2019) The American Association for the Advancement of Science. (**c**) Jellyfish-like motion due to the responsive characteristic of the structured M-PMC. Adapted from [229]. (**d**) Prompt object capturing and releasing with response in milliseconds. Adapted with permission from [230]. Copyright (2018) Springer Nature. (**e**) On-demand release of substances included in a M-PMC capsule. Adapted with permission from [231]. Copyright (2021) The American Association for the Advancement of Science. (**f**) A magnetic sensor is capable of tracking hand motion. Adapted from [232] with permission. Copyright (2020) John Wiley and Sons. (**g**) Tactile sensor unit based on M-PMCs. Adapted from [233]. (**h**) Reconfigurable flexible electronics of M-PMC reshaping. Adapted from [234] with permission. Copyright (2021) John Wiley and Sons.

Versatile applications of the M-PMCs have been demonstrated. By incorporating an array of magnetic-particle-soft-matrix pillars into flat/curved substrates, Gu et al. achieved programmable locomotion of the structured substrates driven by a metachronal magnetic field [228]. By designing the patterns and curvatures of the pillars, specified motion can be encoded into the structured substrates. The substrate can thus mimic the crawling motion millipede, as shown in Figure 10a. However, locomotion does not necessarily need complex structures. Kim et al. showed that complex but accurate locomotion of M-PMCs can be obtained with simple structures like a continuous thread [214], as shown in Figure 10b. The thread can be easily fabricated by extrusion or injection molding due to the geometric simplicity. The reduced fabrication cost can greatly benefit mass production. Because the response of M-PMCs to the driving field shows little lag, propulsion in an aqueous environment is also possible. Ren et al. demonstrated that the structured M-PMC mimics the motion of jellyfish because the structured material controls the flow [229]. In Figure 10c, snapshots of different states during the motion cycle are shown. Such quick responses also can be harnessed to achieve high-speed object manipulation. By locally patterning the direction of the magnetic particles, Kim et al. showed prompt object capture and release, as in Figure 10d [230]. With optimized structural design, extremely useful dedicated capturing/releasing functions can be achieved. For example, controlled release of functional substance (e.g., drugs) from within capsules can be activated upon demand, as shown in Figure 10e [231]. M-PMCs are also valuable as sensing devices because the magnetic behavior enables passive application with few power limitations. A magnetic sensor is capable of tracking hand motion via an accurate record of the electric signal of the sensor, as in Figure 10f [232]. Because of the stacked structure of the sensor, the magnetoelectric signal is amplified. The application of M-PMCs as the tactile sensor is preferable because continuous sensing offers better human–machine interaction. Shown in Figure 10g, the tactile sensor unit is safer to operate by humans due to the compliance of the M-PMC [233]. More functions can be integrated into the M-PMC devices by introducing different units or components. Qi et al. showcased fully integrated flexible electronics that can be shaped under the magnetic field, as in Figure 10h [234].

### 4.2. Shape-Memory Polymer

Structural transformation in shape-memory polymers (SMPs) is controlled by an external stimulus such that different configurations can be adopted according to requirements mandated by specific applications. Designed structures based on manipulation are potentially valuable in different applications. For example, it has been a recent trend to apply coronary stents based on SMPs in medical implantation instead of metals because of both the lower cost and higher tunability [235,236]. The higher tunability of SMPs results from diverse and versatile polymer chemistry, compared with metals. In addition, the densities of SMPs are usually lower than those of shape-memory alloys (SMAs), making the former more favorable in lightweight designs. The functional differences between SMPs and SMAs can be attributed to the different molecular transition mechanisms of the two materials. SMAs, regardless of the type, are metals whose shape-memory effects rely on the phase transition based on changes of atomistic crystal structure of the materials. On the other hand, the shape-memory effects of SMPs are based on diverse transition mechanisms, as discussed below. Therefore, SMPs permit versatile functionalities and flexible design strategies.

Compared with metal, transition/switching mechanisms in polymers can be diverse and versatile. A common functional mechanism in SMPs is based on thermophysical phase-transition of the polymeric constituents, e.g., the glass-to-melt transition characterized by the glass transition temperature, $T_{g}$ [12]. Over a range of temperatures above $T_{g}$ but below the boiling point or degradation temperature, the material without crosslinks behaves like a free-flowing viscous liquid. However, the viscosity increases dramatically as the temperature decreases. When the temperature approaches or goes below $T_{g}$, the material behavior changes from viscous and rubbery to brittle. Such a change is due to the frozen microstructure of the material, which prevents the diffusion of molecules. Conceptually, a type of SMP is achieved by the following: it starts with an amorphous rubbery state above $T_{g}$ such that it is intrinsically homogeneous. If deformation is applied in this state, the material readily changes shape. If the temperature is maintained above $T_{g}$, the changed shape will tend to return to the undeformed state. However, if the temperature is efficiently cooled down below $T_{g}$ in the unrelaxed state, the deformation will become locked. Upon re-heating the locked material, the deformation can be relaxed again, and the material returns to the undeformed state. Another example of SMP application is through the transition of semi-crystalline to the melt state, as characterized by the melting temperature, $T_{m}$. This mechanism is similar to the one described above, with $T_{g}$ replaced by $T_{m}$. In many applications, SMPs operate through reversible switching phenomena, which enables controllable phase transition of the SMP microstructures within different states and thus facilitates the shape transformation. For example, photosensitive constituents in the polymer material can be used to drive the shape-memory (SM) transformation. In such a design, photo-reversibly reactive molecules are grafted onto the polymeric matrix. The molecules undergo a transition between states when exposed to UV light of material-specific frequencies, and the bulk material experiences a resulting change in shape [237]. Another example of a switching mechanism is via physical association/dissociation of functional groups, like hydrogen bonding and metal–ligand coordination [238]. Such processes are triggered by thermal stimulus-based activation-like mechanisms. SM transformation can also be driven by bond-exchanging reactions. For example, some polymer systems with disulfide bonds as the switch mechanism can lead to SM transformation. With thiol components incorporated into the cellulose acetate backbones, the redox state of mercapto groups was successfully changed by exposure to dimethyl sulfoxide solution [239]. Isomerization is another important switching mechanism. For example, trans–cis–trans isomerization of azobenzene, as chromophores, was grafted into different polymeric networks [240]. When the hybrid networks were subject to light, isomerization was triggered, which yielded SM transformation.

**Figure 11:**
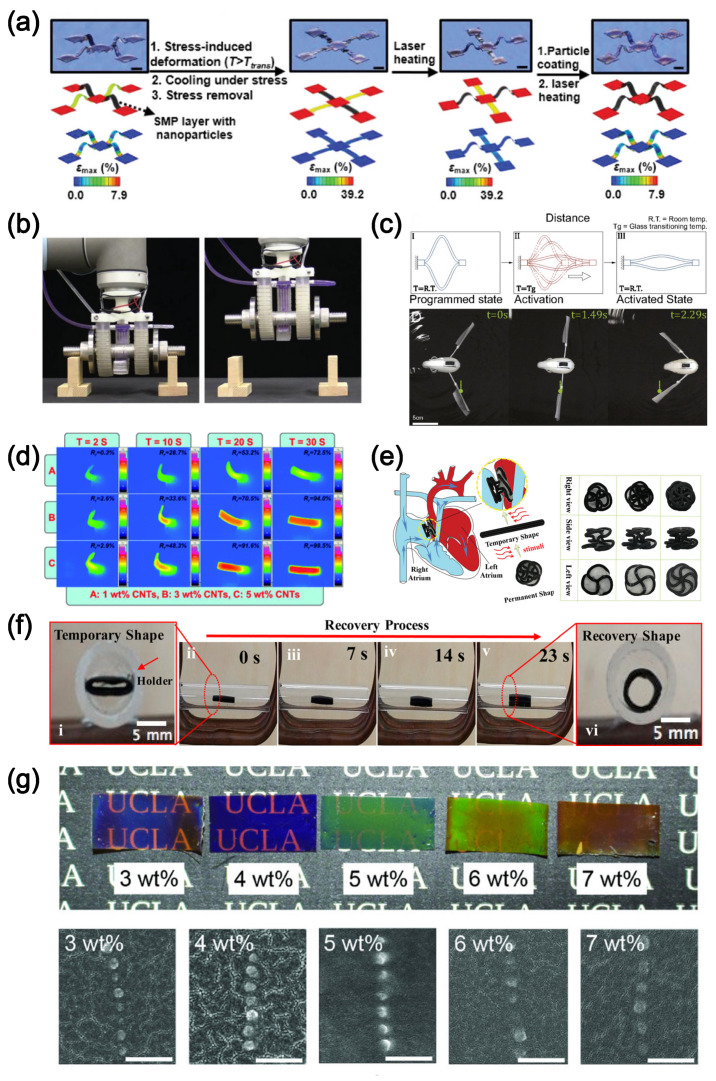
Application of SMP. (**a**) 3-D morphing by SMP. Adapted from [241] with permission. Copyright (2018) John Wiley and Sons. (**b**) Dumbbell lifting actuator based on SMP. Adapted from [242] with permission. Copyright (2019) John Wiley and Sons. (**c**) The motion of the untethered robot with mechanically bi-stable elements. Adapted from [243] with permission. (**d**) SMP mixed with CNT that raises the temperature by microwave absorption. Adapted from [244] with permission. Copyright (2013) Royal Society of Chemistry. (**e**) Occlusion device in temporary and permanent states. Adapted from [224] with permission. Copyright (2019) John Wiley and Sons. (**f**) Magnetoelastic SMP activated inside a tube. i and vi are the side-views. ii to v are time-dependent snapshots showing the activated motion. Adapted with permission from [245]. Copyright (2017) American Chemical Society. (**g**) Color-change SMP composite. Color changes under mechanical deformation. Adapted from [246] with permission. Copyright (2018) John Wiley and Sons.

The morphing properties of SMPs have been utilized to achieve actuation, even at the micrometer scale. Wang et al. designed different structures of SMPs along with their filled composites with dimensions of a few hundred micrometers and obtained 3-D morphing with a standard photolithography method [241]. The schematic for designing the structure is shown in Figure 11a. Since the transformation of most SMPs relies on the change in temperature and photo-reaction, the application of SMPs is more flexible than the M-PMCs, for which magnetic stimulus resources are usually costly and limited. Inclusion of heat circuits and finger-like actuation units with high load capacity to lift a dumbbell is demonstrated in Figure 11b [242]. The state-dependent actuation, along with structural design, has great application potential. For example, Chen et al. proposed SMP structures that can harness mechanical instability and control the directional motion of untethered robots, as in Figure 11c [243]. The robot requires an initial heating-up process for the prepared state. However, such a process can be achieved remotely if appropriate filler particles are introduced. For example, SMPs mixed with carbon nanotubes (CNTs) can be heated up by the absorption of microwaves, as in Figure 11d [244]. Combined with other active mechanisms, like magnetoelasticity, functional SMP devices have the advantage of shape morphing depending on the transformation state. For instance, an occlusion device for congenital heart diseases, based on SMPs with magnetic filler particles, can be implanted in a compact state but later be transformed to the deployed state, through triggering by the magnetic response of the material [224]. Importantly, the device retains structural function because of the state transformation of the SMP material, even with the magnetic stimulus removed, as in Figure 11e. The device is also biodegradable. Similar magnetoelastic SMP structures can be used in other applications involving confined space. Wei et al. fabricated functional structures, with direct write, that can be operated inside a tube [245], as in Figure 11f. With appropriate fillers, the photonic properties of the composites can be altered as well. Xie et al. fabricated reusable color-displaying composites by controlling the alignment of the magnetic filler particles [246]. Such composites change colors due to the change of the spacing of the particles under mechanical deformation, as shown in Figure 11g.

### 4.3. Self-Healing Polymer

Self-healing polymers (SHPs) form another fascinating class of smart polymer systems that can fully or partially recover degraded or damaged material properties [247]. The recoverable property is advantageous in applications where it is expensive and/or difficult to replace the damaged materials. Self-healing mechanisms are categorized into physical and chemical types, or combinations of both, including diffusion of constituents [248], phase effect [249], and physical association/dissociation processes [250]. The diffusion process is particularly relevant to thermoplastic polymer materials under operation conditions above $T_{g}$. Thermal diffusive motion of the polymer molecules at these temperatures enables altering of the microstructural states and can lead to recovery to some extent. If the material is in a glassy state, recovery of the damage can be achieved by melting and welding. Microscale mixing of semi-compatible or incompatible phases due to the competition between mixing energy and entropy can, in some cases, facilitate the repair of the damaged interface. Associative chemistry like hydrogen bonds, guest–host chemistry, and metal–ligand coordination can be used for self-healing.

**Figure 12:**
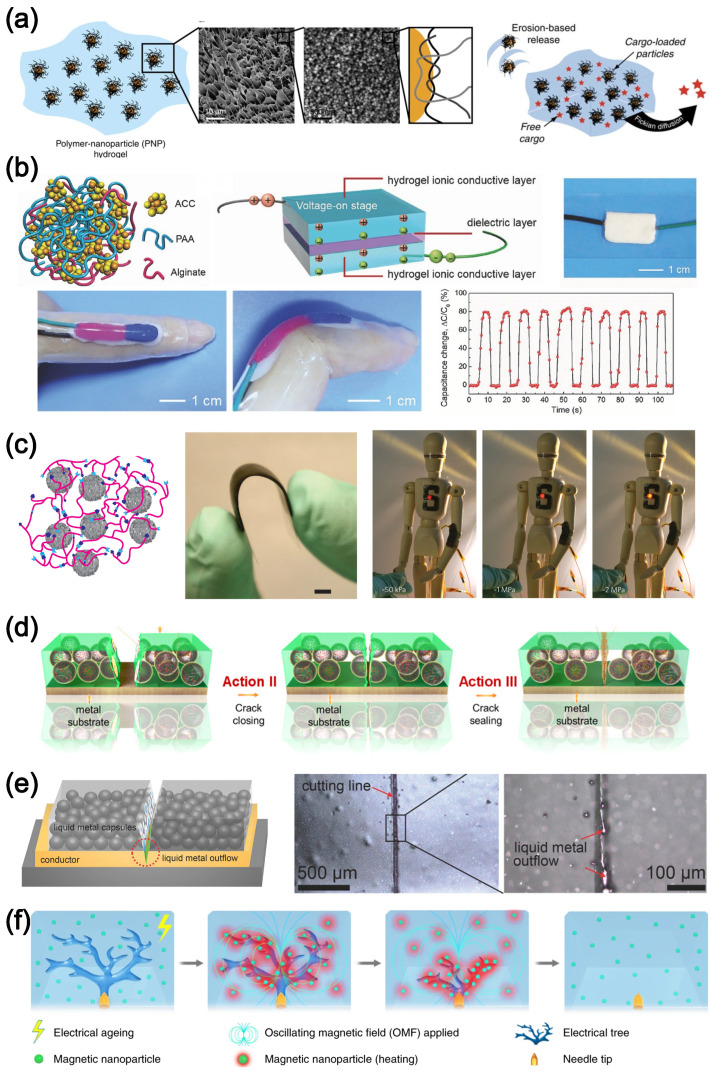
Application of SHP. (**a**) Self-healing hydrogel with a dual mechanism to deliver hydrophilic and hydrophobic therapeutics. Adapted from [251] with permission. Copyright (2015) Springer Nature. (**b**) Nanoparticles as physical crosslinkers in the hydrogel facilitate the self-healing process of a reliable flexion sensor. Adapted from [252] with permission. Copyright (2017) John Wiley and Sons. (**c**) Flexion and tactile sensing application. Adapted from [253] with permission. Copyright (2017) Springer Nature. (**d**), Adapted from [254], and (**e**), adapted from [255] with permission (Copyright (2018) John Wiley and Sons.), show examples of self-healing of coating layer by SMP contraction and released protecting or recovery substances. (**f**) Magnetic nanoparticles excited under stimulation generate heat and heal the crack matrix. Adapted from [256] with permission. Copyright (2018) Springer Nature.

Because of the unique properties of SHPs, they are very suitable as functional material platforms. Filled with surface-modified NPs that can bind therapeutic molecules, Appel et al. proposed SHP materials with a dual-release method to implement drug delivery for both hydrophilic and hydrophobic therapeutics at the same time [251]. Beyond introducing reinforcement to the SHP, the filler particles can also be released as cargo. The dual delivery mechanism is shown in Figure 12a. Lei et al. proposed a self-healing hydrogel that is suitable for sensing applications [252]. The filler particles serve as physical crosslinkers that permit the healing process under room temperature conditions. The instantaneous repairing capability suggests a reliability sensing unit, as shown in Figure 12b. Such a sensing mechanism is also feasible for other applications. Flexion and tactile detection with a similar composite were demonstrated by Tee et al. [253], as shown in Figure 12c. Additional self-healing strategies have also been explored in the literature. For example, an SMP that is intrinsically non-self-healing is filled with microbubbles that include corrosion inhibitors as a self-healing coating to protect metal-containing materials [254]. When damage to the surface coating exposes the metal components to a corrosive environment, as in Figure 12d, the microbubbles are ruptured and the protective substance is released to cover the damaged area. Additional heating can induce shape transformation of the SMP matrix such that the opening shrinks to shield the substance filling the damage. Similar strategies apply to other protecting and recovering substances. By mixing the polymer matrix with microbubbles encapsulating liquid metal, Chu et al. showed that upon scratching of the composite the liquid metal leaks from damaged capsules and creates a protective cover at the location of the scratches [255]. The comparison before and after recovery is shown in Figure 12e. Another interesting example of a non-intrinsic self-healing filled matrix involves the addition of magnetic particles by Yang et al. to a polymer used for electrical insulation [256]. Repeated electrical load over time tends to form microcracks and degrade the insulation property of such material. As a remedy, superparamagnetic NPs are included in the polymer matrix. An alternating magnetic stimulus can be used to excite the NPs whose motion generates sufficient heat to wield the microcracks, as shown in Figure 12f.

### 4.4. Constitutive Modeling for Smart Materials

To consider the magnetic response of materials in a constitutive model, the magnetoelastic contribution must be included in the free energy expression. For this, one defines the residual remanence, ${\bar{B}}_{r}$, at point x in the current configuration, mapped from the reference configuration function, $B_{r}\left(X\right)$. The free energy density in the reference volume is then given by Equation (15),

(15) $$W_{magnetic}=-\frac{1}{\mu_{0}}F{\bar{B}}_{r}\cdot B_{ext}$$

where $\mu_{0}$ is the permeability and $B_{ext}$ the applied magnetic field [65,257]. The solution to the boundary problem, $W_{magnetic}$, can be obtained by finite element or particle-based methods [258]. However, in such a model, $W_{magnetic}$ is assumed to be continuous while the magnetic response is governed by intrinsic discrete particles. Such an approach is appropriate when the particles are evenly dispersed in the matrix and no macroscopically nonuniform secondary or tertiary agglomerate structure or polymer–particle interface delamination occurs [259]. In the case of the latter, it can become important to include additional descriptions accounting for the microscopic structure. Inclusion of the Mullins and the Payne effects could also be necessary to model inelastic behavior.

For constitutive modeling of the shape memory effect, the transition between states, as described phenomenologically in Figure 7a, can be used to construct the free energy density function. For example, Mao et al. developed a thermoviscoplastic model that considers an anisotropic shape memory effect whose time-dependence is attributed to the viscoplasticity of the matrix, and the anisotropy comes from the additive fibers [260,261]. However, such a model involves a few fitting parameters that are difficult to interpret physically. If one needs to incorporate microscopic structural information into the constitutive equations, care must be taken to model the constituents correspondingly. For example, the shape-memory effect related to the viscoplasticity of a semi-crystalline polymer can be modeled quantitatively by a micro-mechanistic model that uses a representative volume element within a multiscale-framework [190]. Different phases of the material need to be modeled explicitly so that their contribution to the bulk free energy density can be monitored. Xiao et al. analyzed shape-memory behavior driven by glass transition by explicitly introducing a nonlinear recovery process [262]. Parameters of the derived model have physical significance related to the glass transition and thermal expansion and they can be interpreted quantitatively. Another example is the chemo-thermo-mechanically coupled viscoelastic model proposed by Mao et al., which links the shape-memory effect to the chemical potential that governs the phase-transition induced by the bond exchange reaction [64].

The time-dependent evolution of damage caused by micrometeorite impacts is also crucial for modeling self-healing processes since many self-healing materials rely on physical and chemical bond-reforming processes to recover strength and surface texture. While phenomenological formulations [263] may be sufficient to model the recovery, physics-driven models are preferable. Differential equations linking the number of reformed bonds and the recovering force are suitable to formulate the process of recovery if the bond-reforming process is described by a kinetic process. For example, Hui and Long modeled the recovery process of the secondary network in a double-network hydrogel by tracking the concentration of the secondary bonds explicitly [264]. The concentration of secondary bonds, through its inclusion in the expression for time-dependent free energy density, W(t), contributes directly to the strength of the network. It has been shown that the time-dependent framework can describe the damage and recovery process for different material systems [63,264,265].

## 5. Discussion and Conclusions

Macroscopic mechanical behavior of filled elastomers clearly depends on the filler–matrix microstructures and dynamics. Significant progress has been achieved in terms of developing phenomenological and mechanistic constitutive models of such materials at the continuum level. Some of these models involve interpretable parameters in terms of underlying physical processes. However, there is still a gap between macroscale and microscale modeling for the filled system, with a few challenges to overcome.

The first challenge is limitation in the experimental probing of the microscopic structural information. For example, it is still difficult to directly observe fracture processes of the polymeric network, especially in bulk, although progress is being made. Ducrot et al. developed systems of multi-network elastomers with chemoluminescent molecules embedded as crosslinkers such that photons are emitted when the molecules break [266]. Although localized damage zones were observed under the photon-detecting camera, the damage process details at the microscopic level remained unclear due to the limited spatial resolution of the camera. A similar limitation was also encountered in another study where mechanophores were embedded in the network [267,268]. Although the bond-breakage process of a single molecule was well characterized, the understanding of the relation between a single bond breakage and bulk fracture of the network was not fully clarified. While molecular simulation can be a powerful tool in probing such dynamics [269], elasticity [270], viscoelasticity [271], and phase-separation behavior [272], it is computationally intensive and usually limited in terms of both spatial and temporal scales of practical interest. To circumvent some of these limitations, a promising approach is the mesoscale modeling technique. In mesoscale modeling, it is critical to select the appropriate temporal and spatial scales as references. For example, in the modeling of vitrimer systems, Wagner and Vernerey set the time unit as the transient bond lifetime of the associative reaction and the length unit as the molecular size of the linear chains [273]. Such a choice of basic units efficiently captures the dynamically reforming network structure and the corresponding viscoelastic properties. In another example, Lamont et al. modeled the slide-ring networks based on a similar philosophy [274]. Instead of modeling the polymer chains with full molecular details, the authors employed free energy functions that are dependent on the length of chains. With a similar focus to the network structure, Zhang et al. showed that the concentration of chains in the network affects the modes of failure [275]. Failures can be in the form of diffused bond breakage, crack nucleation, avalanches, and percolation of bond breakage [276]. Mesoscale modeling offers promise as a systematic method to link microscopic structural information obtained from experimental characterization or molecular simulation to the macroscopic bulk properties of both unfilled and filled networks.

The other challenge is the complexity of involving the diverse chemistry of polymeric materials. Although the commonly used materials discussed in Section 2 are well studied, it takes time and effort to understand emerging new material systems. For example, there are many thousands of molecules as potential candidates for the application of dielectric insulation, thermal conduction, energetic materials, and drug development [277,278,279,280,281]. It is difficult and inefficient to manually screen such a vast number of candidates to select optimized candidates for product development. Machine learning (ML) assisted protocols are extremely valuable in guiding the screening processes. For example, to select materials with the desired glass transition temperature, $T_{g}$, for the SMP or SHP, it is very efficient to estimate values of $T_{g}$ though different ML algorithms [282]. ML techniques can also be used to facilitate the design of copolymers with targeted properties like $T_{g}$ and viscosity, η, to meet different criteria [280,283].

Data-driven techniques are also important in terms of constitutive modeling. Potentially, intrinsic multiscale and multitask data-driven techniques can be used to directly link the microscopic structure information from molecular and micromechanical simulation to the continuum such that even complex problems like contact and shear banding that are common in the granular material system and filled elastomer can be understood quantitatively in a unified framework [284]. Although the model parameters of the mechanistic and physics-driven constitutive models are connected to the chemistry and microscopic properties of the materials, parameter-fitting and model-calibration processes are sometimes necessary for the application of the models. For example, in the nonaffine model by Davidson and Goulbourne [53], the hyperelastic stiffness of a model elastomer is decomposed into crosslinked and entanglement components. The values of the entanglement modulus, $G_{e}$, for crosslinked networks may not be the same as the values of the corresponding uncrosslinked precursors that have been quantified extensively [285]. Therefore, it requires additional efforts to calibrate the material-specific parameter values in the application of the nonaffine model [286]. Such calibrations are labor-intensive optimization processes, which can be circumvented by data-driven techniques [287].

One type of data-driven technique is the neural network (NN)-based method that has been developed over three decades [288]. The most straightforward implementation of the NN-based method is to use the applied (history of) strain as input and the stress response as output such that stress–strain relations, as material properties, can be learned by the NN directly [289,290]. Nonetheless, such implementation requires substantial amounts of training data to achieve convergence of the NN because a typical stress–strain curve includes tens of or even hundreds of data points. Therefore, compression of the training data is the first important aspect before feeding the data to the NN training processes. For example, the deformation gradient, F, or the corresponding invariants of F, instead of the applied strain, can be used to decrease the dimensionality of the NN and thus accelerate the training processes [291,292,293,294]. Mathematical operations like principal component analysis (PCA), singular value decomposition (SVD), and Isomap can also be used to facilitate the dimensional reduction [295,296,297]. Another important aspect in the development of the NN-based method is to enforce established knowledge of physics. For example, the free energy density function, *W*, or other governing equations can be used as the loss function (optimization goal) of the NNs to explicitly enforce the physical constitutive laws rather than optimizing the model parameters [298,299,300]. It is elegant to embed the physical laws in the loss function such that no presumptive functional forms for additional constraints are required. For instance, incompressibility can be enforced by including material compressibility in the loss function rather than a numerical method like the Lagrange multiplier that is usually needed for theoretical modeling of elastomers [301]. In addition to NN-based methods, other data-driven techniques are also valuable in constitutive modeling. For instance, it is much easier to conduct one-dimensional mechanical tests, like uniaxial tensile tests, than complex loading conditions. However, it requires care to calibrate generally three-dimensional constitutive models using the one-dimensional data from the mechanical tests. Tang et al. developed data-driven protocols that harness one-dimensional data to predict the corresponding three-dimensional stress–strain behavior without assigning analytical constitutive relations [302,303,304]. Another example is the unsupervised learning protocol proposed by Flaschel et al. that leverages the least absolute shrinkage and selection operator (LASSO) to augment the optimization processes to study hyperelastic material laws [305]. The unsupervised protocol requires only one pair of stress–strain data samples to uncover the underlying constitutive law.

Significant attention is being paid to developing models that are interpretable [306]. In addition to the construction of loss functions that enforce desired physical laws, other methods are also available to develop interpretable data-driven models. For instance, specific sub-NNs can be designated for different invariants of F instead of using one NN for all of the invariants such that the relations between the invariants and the free energy density, *W*, can be distinguished quantitatively [307]. Similarly, algebraic or differential operations can be assigned to specific neurons by choosing appropriate activation functions such that the output of each operation can be understood quantitatively [308,309]. However, the algebraic or differential operations included in the (neurons of) NNs rely on a priori selection of functional forms and thus could bias the physical laws that they are supposed to represent. To exclude possible bias, the functional forms of the operations can be studied by symbolic regression [310].

In this work, we discussed the mechanical properties of unfilled and filled elastomers from the perspectives of continuum and microscopic properties of the materials. The mechanical properties are determined by the constituent polymers and filler particles. The roles of the filler particles in the systems are not limited to reinforcement. The microstructure of the particles, like aggregation and agglomeration, directly affects the reinforcement, hyperelastic, and inelastic behavior of the PMC. We introduced different phenomenological and physics-driven constituent models and presented their connection to the microscopic structural information. In addition, we discussed filled systems used as smart materials for different applications, and found that the functions of smart materials are partially or fully determined by the properties of the filler particles. Different magnetoelastic, shape-memory, and self-healing material systems were discussed, with relevance to a variety of applications. Aspects of constitutive modeling for these smart materials were also provided. To overcome the knowledge gap between microscopic properties and macroscopic bulk properties of the material systems, it is important to include the mesoscale modeling method as a bridging element and harness the ML techniques to facilitate fast material screening processes.
